# Single‐cell analysis uncovers high‐proliferative tumour cell subtypes and their interactions in the microenvironment of gastric cancer

**DOI:** 10.1111/jcmm.18373

**Published:** 2024-06-19

**Authors:** Wenjia Zhang, Xiaojing Wang, Jiaxing Dong, Kai Wang, Wanju Jiang, Chenchen Fan, Haitao Liu, Lihong Fan, Lei Zhao, Guoshu Li

**Affiliations:** ^1^ Department of Respiratory Medicine, Shanghai Tenth People's Hospital Tongji University School of Medicine Shanghai China; ^2^ Department of Rheumatology and Immunology, Tongren Hospital, School of Medicine Shanghai Jiao Tong University Shanghai China; ^3^ Department of Gastrointestinal Surgery, Shanghai East Hospital, School of Medicine Tongji University Shanghai China; ^4^ Shanghai YangZhi Rehabilitation Hospital (Shanghai Sunshine Rehabilitation Center), School of Medicine Tongji University Shanghai China

**Keywords:** cell proliferation, gastric cancer, prognosis, single‐cell RNA‐sequencing, treatment

## Abstract

Gastric cancer (GC) remains a prominent malignancy that poses a significant threat to human well‐being worldwide. Despite advancements in chemotherapy and immunotherapy, which have effectively augmented patient survival rates, the mortality rate associated with GC remains distressingly high. This can be attributed to the elevated proliferation and invasive nature exhibited by GC. Our current understanding of the drivers behind GC cell proliferation remains limited. Hence, in order to reveal the molecular biological mechanism behind the swift advancement of GC, we employed single‐cell RNA‐sequencing (scRNA‐seq) to characterize the tumour microenvironment in this study. The scRNA‐seq data of 27 patients were acquired from the Gene Expression Omnibus database. Differential gene analysis, Gene Ontology, Kyoto Encyclopedia of Genes and Genomes and Gene Set Enrichment Analysis were employed to investigate 38 samples. The copy number variation level exhibited by GC cells was determined using InferCNV. The CytoTRACE, Monocle and Slingshot analysis were used to discern the cellular stemness and developmental trajectory of GC cells. The CellChat package was utilized for the analysis of intercellular communication crosstalk. Moreover, the findings of the data analysis were validated through cellular functional tests conducted on the AGS cell line and SGC‐7901 cell line. Finally, this study constructed a risk scoring model to evaluate the differences of different risk scores in clinical characteristics, immune infiltration, immune checkpoints, functional enrichment, tumour mutation burden and drug sensitivity. Within the microenvironment of GC, we identified the presence of 8 cell subsets, encompassing NK_T cells, B_Plasma cells, epithelial cells, myeloid cells, endothelial cells, mast cells, fibroblasts, pericytes. By delving deeper into the characterization of GC cells, we identified 6 specific tumour cell subtypes: C0 PSCA+ tumour cells, C1 CLDN7+ tumour cells, C2 UBE2C+ tumour cells, C3 MUC6+ tumour cells, C4 CHGA+ tumour cells and C5 MUC2+ tumour cells. Notably, the C2 UBE2C+ tumour cells demonstrated a close association with cell mitosis and the cell cycle, exhibiting robust proliferative capabilities. Our findings were fortified through enrichment analysis, pseudotime analysis and cell communication analysis. Meanwhile, knockdown of the transcription factor CREB3, which is highly active in UBE2C+ tumour cells, significantly impedes the proliferation, migration and invasion of GC cells. And the prognostic score model constructed with CREB3‐related genes showcased commendable clinical predictive capacity, thus providing valuable guidance for patients' prognosis and clinical treatment decisions. We have identified a highly proliferative cellular subgroup C2 UBE2C+ tumour cells in GC for the first time. The employment of a risk score model, which is based on genes associated with UBE2C expression, exhibits remarkable proficiency in predicting the prognosis of GC patients. In our investigation, we observed that the knockdown of the transcription factor CREB3 led to a marked reduction in cellular proliferation, migration and invasion in GC cell line models. Implementing a stratified treatment approach guided by this model represents a judicious and promising methodology.

## 1 INTRODUCTION

Gastric cancer (GC) is the fifth most prevalent malignancy worldwide.^1^ For the treatment of GC, in the early stages, surgical resection stands as the primary intervention, either with or without neoadjuvant therapy. Conversely, chemotherapy assumes a primary role during the middle and advanced stages, sometimes supplemented by immunotherapy or targeted inhibitors like trastuzumab.^1^ Unfortunately, the rapid progression of GC often results in diagnosis occurring primarily in the middle and late stages of the disease. This lamentable circumstance contributes to the alarming mortality rates associated with GC, warranting its grim standing as the third leading cause of cancer‐related fatalities worldwide.^2^ A study published in 2022 predicted that the number of new GC cases worldwide will increase by 62% to 1.77 million by 2040.^3^

The unrestrained proliferation of cancerous cells forms the foundation for the swift emergence and progression of GC. Cell proliferation is one of the important physiological functions of cells, and this process must be precisely regulated. Even the slightest deviations can disrupt the control mechanisms, propelling normal cells into a state of unbridled growth, ultimately resulting in the onset of GC. The proliferation of tumour cells is mainly related to the imbalance of mitosis, including the imbalance of cell cycle and apoptosis.^4^, ^5^ The disorders of these biological processes are regulated by the complex tumour microenvironment (TME). GC tissue is not a mass composed of single cancer cells, but a microecological structure, including various types of stromal cells, immune cells and various cytokines.^2^ These complex factors lead to huge inter‐patient and intratumoral heterogeneity in GC. Therefore, an in‐depth understanding of the inherent characteristics of GC is the only way to improve the survival rate of patients and promote the development of tumour precision medicine.

The mechanism underlying tumour genesis and progression is intricate and dynamic, yet at its core, it manifests as a malignant proliferative disorder spurred by genetic mutations. Only through genetic analysis can we acquire the corresponding repertoire of variations within tumour cells. Single‐cell RNA‐sequencing (scRNA‐seq) can detect multiple variation forms of hundreds of genes in a short time, which greatly accelerates our research in the field of tumour molecular biology. This technique stands as one of the paramount approaches for assessing the molecular markers of tumours, including tumour mutation burden (TMB), microsatellite instability, gene point mutations, insertions, deletions, copy number variations and gene fusions. It grants us an unparalleled opportunity to delve into the ecological landscape of tumours. Furthermore, scRNA‐seq holds the potential to offer invaluable clinical guidance for monitoring disease progression, prognostic risk assessment and the identification of therapeutic targets.

In this investigation, we undertook the reconstruction of the GC microenvironment through the application of scRNA‐seq. By scrutinizing the scRNA‐seq data, we unveiled a distinctive cell population defined by the human ubiquitin‐conjugating enzyme E2C (UBE2C) and an associated biological target linked to the accelerated advancement of the GC. Subsequently, employing bulk RNA‐sequencing, we devised a pertinent risk model and explored its correlation with immune infiltration and therapeutic drug utilization. Through our endeavours, we aspire that this study may provide personalized diagnosis and treatment strategies and medication guidance for GC patients, in order to improve the prognosis and reduce the mortality of patients.

## 2 METHODS

### 2.1 Identification of cell types

The cell clustering process was conducted using the ‘FindClusters’ and ‘FindNeighbors’ function. According to the CellMarker database (http://xteam.xbio.top/CellMarker/) and the typical markers of cell subsets agreed from previous studies, combined with the automatic annotation capability of ‘Single R’, the cells were manually annotated. To identify differentially expressed genes (DEGs) within distinct cell subsets, the ‘FindAllMarkers’ function was employed, with min.pct and min.diff.pct was set to 0.25 and logfc.threshold was set to 0.25. Furthermore, in order to investigate the heterogeneity of the tumour cells, they were subjected to a re‐clustering process. Annotation of the tumour cell subpopulations was accomplished by utilizing tumour cell marker genes.

### 2.2 Functional enrichment analysis

Gene Ontology (GO) and Kyoto Encyclopedia of Genes and Genomes (KEGG) enrichment analyses were performed on DEGs of different cell types using ClusterProfiler R package (4.6.0 version)^6^, ^7^, ^8^ Gene Set Enrichment Analysis (GSEA) software (version 4.1.0) was downloaded from the GSEA website (http://software.broadinstitute.org/gsea/msigdb) for analysing the pathway gene sets downloaded from the MSIGDB database (https://www.gsea‐msigdb.org/). In order to analyse the distinctions between different risk groups within the bulk data, the DESeq2 R package was employed, establishing the threshold as |logFC| > 2 with *p*‐value less than 0.05.

### 2.3 Identification of malignant cells by inferCNV

In order to distinguish malignant cells from non‐malignant cells, the initial copy number variation (CNV) level of each region was estimated from the disturbance of chromosome gene expression using inferCNV R package (https://github.com/broadinstitute/inferCNV/wiki). Taking endothelial cells as a reference, the CNV spectrum of cell subsets was calculated by inferCNV algorithm, and the epithelial cell subsets showing significant CNV spectrum were defined as malignant cells.

### 2.4 Pseudotime analysis

Cell stemness was evaluated through the utilization of the CytoTRACE R package (version 0.3.3),^9^ allowing for speculative inference of the chronological sequence of cell differentiation.

Monocle R package (version 2.24.0) was used to analyse the pseudotime trajectory of tumour cells. Dimension reduction was achieved using the UMAP method, with subsequent visualization facilitated by the ‘PLOT_CELL_TRACTURE’ function. The different cell subpopulations were then sorted based on their pseudotime order. Genes exhibiting synchronized changes along the pseudotime trajectory were identified and represented through the use of a pseudotime heatmap.^10^

The ‘getlineage’ function in Slingshot R package (version 2.6.0) was utilized to fit a minimum spanning tree to infer cell developmental lineages. The ‘getCurves’ function was used to calculate the expression levels of different lineages during the fitting time.

### 2.5 Cell communication analysis

The CellChat R package (version 1.6.1)^11^ was used to infer complex cell‐to‐cell interactions and to establish regulatory networks based on ligand‐receptor levels. It employs the ‘netVisualDiffInteraction’ function to represent the difference in communication strength between cells, and the ‘IdentifyCommunicationPatterns’ function to estimate the number of communication patterns. A significant threshold of 0.05 (*p*‐value) was used. Circle plots, bubble plots and violin plot were used to visualize the incoming and outgoing signals from all cells. We focused on the analysis of cellular communication and related pathways between tumour cells and other cells, and explored how signal pathways can be coordinated among various cell types.

### 2.6 Scenic analysis

In order to investigate the top 5 transcription factors (TFs) with the most significant changes in the expression of each tumour cell subgroup, the pySCENIC R package (version 0.10.0) in Python (version 3.7) was used for single‐cell regulatory network inference and clustering analysis. Firstly, GRNBoost was employed to identify potential target genes for each TFs. After that, DNA‐motif analysis helped identify potential direct binding targets. Finally, the activity of regulon in the cells was scored by AUCell, and the top 5 TFs with the highest score were selected. The human gene‐motif ranking is obtained from https://resources.aertslab.org/cistarget/.

### 2.7 GC prognostic model construction

The expression values of patient‐characterized genes in the TCGA_GC cohort were extracted and used to perform the univariate Cox regression analysis to screen for potential GC prognosis‐related predictor genes. LASSO Cox regression analysis (glmnet, version 4.1‐6) was used to further screen genes to avoid the multicollinearity of these genes. The risk coefficient of each gene was calculated by multivariate Cox regression analysis and the prognostic risk score model was established. Risk scores of the prognostic risk score model (*X*: coefficient, *Y*: gene expression level) = $\sum_{i}^{n}X_{i}\times Y_{i}$. Based on the median risk score, patients were categorized into high‐risk and low‐risk groups for further analysis. Survival analysis was performed in the TCGA‐GC cohort using Kaplan–Meier formula in Survival R package (version 3.3‐1), and the survival curve was visualized using the ‘ggsurviplot’ function^12^ to compare outcomes of patients in different risk groups. The Time ROC R package (version 0.4) was used to plot the change in the time‐varying area under the curve of ROC (AUC), to assess the predictive performance of the prognostic risk score model. Multivariate Cox regression analysis was used to evaluate the relevant clinicopathologic factors to confirm the role of prognostic risk scores as an independent prognostic factor. The Nomogram of Cox regression model was established and the 1‐, 3‐ and 5‐year overall survival (OS) rates of GC patients in TCGA cohort were predicted. Plot calibration plots of survival, ROC curves, and the C‐index to evaluate the Nomogram predictive ability.

### 2.8 Cell culture

Both AGS cell line and SGC‐7901 cell line were obtained from American type culture collection. The two cell lines were cultured in F12K medium and PRMI1640 medium containing 10% fetal bovine serum (Gibco BRL, USA) and 1% streptomycin/penicillin, respectively, and cultured under standard conditions (37°C, 5% CO_2_, 95% humidity).

### 2.9 Cell transfection

CREB3 knockdown was achieved using small interfering RNAs constructs (GenePharma, Suzhou, China). The transfection process adhered to the instruction steps provided by Lipofectamine 3000RNAiMAX (Invitrogen, USA). Cells were seeded in 6‐well plates at 50% confluence and subsequently transfected with negative control (si‐NC) and knockdown (Si‐CREB3‐1 and Si‐CREB3‐2) constructs. Each transfection was carried out employing Lipofectamine 3000RNAiMAX (Invitrogen, USA). The siRNA sequences are listed in Table S1.

### 2.10 Cell viability assay

The cellular vitality of AGS cells and SGC‐7901 cells was evaluated post‐transfection through the implementation of CCK‐8 assay. A suspension of cells was introduced into a 96‐well plate, with a density of 5 × 10^3^ cells per well and incubated for a duration of 24 h. After adding 10 μL of CCK‐8 labelling agent (A311‐01, Vazyme) to each well, the plates were incubated at 37°C for 2 h away from light. Cell viability was assessed by detecting the absorbance at 450 nm using an enzyme marker (A33978, Thermo) sequentially on days 1, 2, 3 and 4. The average OD values were calculated and plotted on a line graph.

### 2.11 5‐ethynyl‐2′‐deoxyuridine proliferation assay

The transfected AGS cells and SGC‐7901 cells were inoculated into 6‐well plates at a density containing 5 × 10^3^ cells per well and cultured overnight. A 2× EdU working solution was prepared in serum‐free medium with 10 mM EdU solution and added to the cell culture medium and incubated at a temperature of 37°C for a duration of 2 h. The medium was subsequently discarded, and the cells were washed with PBS before being fixed with a 4% paraformaldehyde solution for a period of 30 min. The cells were then treated with glycine (2 mg/mL) and 0.5% Triton X‐100 for 15 min. Cells were incubated with 1 mL of 1× Apollo and 1 mL of 1× Hoechst 33342 for 30 min at room temperature. The assessment of cell proliferation was carried out utilizing fluorescence microscopy.

### 2.12 Wound healing

The transfected cells were inoculated into 6‐well plates and cultured until the cell density reached 95%. Firstly, use the tip of a 200 μL sterile pipette to cross the cell layer in a straight line in the cell culture wells and then gently rinse the culture wells with PBS. The medium was changed to continue the cell culture. Photographs of the scratch at the same location at 0 and 48 h were collected and the width of the scratch was measured.

### 2.13 Transwell experiment

Prior to the experiment, cells were starved in serum‐free medium for 24 h. Subsequent to the treatment with the addition of matrix gel (BD Biosciences, USA), the cell suspension was added to the upper chamber containing Costar and the serum medium was added to the lower chamber. The cells were incubated in the incubator for 48 h. After incubation, the cells were fixed with 4% paraformaldehyde and stained with crystal violet to assess the invasive ability of the cells.

### 2.14 Western blotting

The transfected cells were cultured until the cell density reached 70%. Cells were lysed with RIPA buffer, supernatants were collected, and centrifuged at 12,000 rpm for 15 min for SDS‐PAGE. After that, proteins were transferred to PVDF membranes, blotted with 5% BSA for 1.5 h at room temperature, incubated with Anti‐CREB3 antibody (ab180119, abcam) at 4°C overnight and then incubated with the corresponding secondary antibody for 1 h. The membranes were visualized by ECL Western Blot substrate.

## 3 RESULTS

### 3.1 Single‐cell RNA‐seq characterization of major cell types in the GC microenvironment

To unravel the complex cellular landscape within the TME of GC, we conducted a comprehensive analysis utilizing a meticulously curated dataset comprising 38 scRNA‐seq samples from 27 GC patients. These 38 samples encompassed various subtypes, including 14 samples of primary tumours exhibiting intestinal characteristics, 6 samples of primary tumours presenting diffuse characteristics, 3 samples displaying a mixed phenotype of both intestinal and diffuse (Mixed) characteristics, 5 samples derived from metastatic sites (2 from stomach metastasis and 3 from peritoneum metastasis) and 10 samples of normal tissue (9 from stomach tissue and 1 from peritoneum tissue). Sample size and clinical characteristics of patients are summarized in Table S2. Following an exhaustive process of quality control and standardization, a collection of 141,187 cells of exceptional quality was ultimately preserved. To identify the major cell types contained in this atlas, we commenced by assessing the canonical marker genes employed in prior research and then examined the highly differentially expressed genes in each cell cluster relative to the other clusters to delineate distinct cellular lineages. Through this meticulous exploration, we finally succeeded in assigning the 141,187 cells to 8 discernible cell types, as elegantly illustrated in Figure S1A,B. These cell types encompassed the likes of NK_T cells, B_Plasma cells, epithelial cells, myeloid cells, endothelial cells, mast cells, fibroblasts and pericytes. To illuminate the multifaceted tapestry of heterogeneity within the GC TME, we embarked upon an exploration to unveil the diverse profiles of cellular composition across different tissue sources and cell cycles (Figure S1B,C). Among these, epithelial cells were predominantly enriched in normal tissues and least in metastatic tissues. Conversely, the realm of intestinal, diffuse, mixed and metastatic tissues blossomed with an abundance of immune cells, while bearing a relatively diminished representation within the realm of normal tissues. Through an analysis of the distribution and scoring of nCount_RNA, nFeature_RNA, S.score, and G2M.score in various cell types, we were able to further clarify the differences between the various cell subpopulations (Figure S1D,E). Notably, B_Plasma cells and epithelial cells exhibited elevated nCount RNA scores, thereby signalling an elevated realm of cellular proliferation fervour.

### 3.2 Characterization of GC tumour cell types identified by scRNA‐seq

**FIGURE 1:**
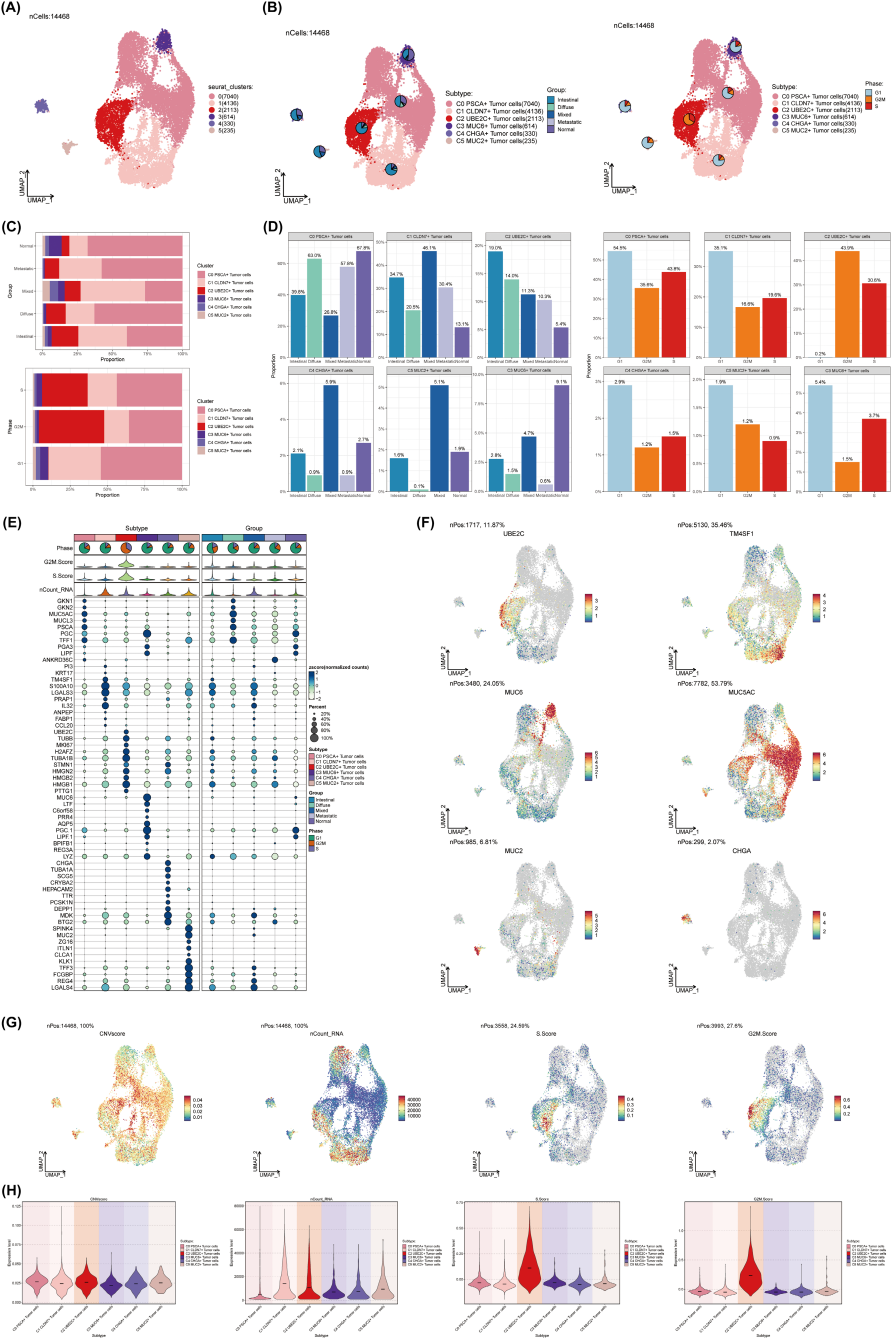
Characterization of GC tumour cell types identified by scRNA‐seq. (A, B) UMAP plots visualization of 6 tumour cell clusters, coloured by cell type. Pie charts showing the tissue origin distribution and cell cycle distribution of different cell types. Left: Group (Intestinal, Diffuse, Mixed, Metastatic, Normal), Right: Phase: (G1, G2M, S). (C, D) Bar plots demonstrating the percentage of different tumour cell types in different tissue types and different cell cycles. (E) Bubble plot demonstrating the differential expression of top 10 marker genes in different tumour cell types and their expression in different tissue types. The size of the bubbles indicates the percentage of gene expression and the colour is based on normalized data. (F) UMAP plots of maker genes in different tumour cell types. (G) UMAP plots demonstrating the distribution of CNVscore, nCount_RNA, S.score and G2M.score in different tumour cell types. (H) Violin plots demonstrating the high and low values of CNVscore, nCount_RNA, S.score and G2M.score in different tumour cell types.

Considering the profound import attributed to neoplastic cells within the TME, our subsequent pursuit entailed the characterization of these cells within the microenvironment of GC. In order to identify epithelial cells with abnormally amplified or missing chromosome copy numbers, we first used inferCNV to analyse the chromosome CNV of epithelial cells with endothelial cells as reference (Figure S2). On the basis of CNV levels, tumour cells were distinguished from epithelial cells. After that, we once more carried out batch effect elimination and quality control on the separated tumour cells. As a result of this meticulous process, the 14,468 tumour cells underwent a comprehensive reclassification into 6 distinct cell types (Figure 1A). After cellular annotation of the 6 tumour cell types based on marker gene expression, we were able to identify the following: C0 PSCA+ tumour cells, C1 CLDN7+ tumour cells, C2 UBE2C+ tumour cells, C3 MUC6+ tumour cells, C4 CHGA+ tumour cells and C5 MUC2+ tumour cells. Their distribution in various tissue sources and cell cycles was shown by UMAP plots and pie charts (Figure 1B). The bar plots presented a more perceptibly expressive portrayal of the distribution of diverse tumour cell types across various tissue compositions and cell cycles. Illustrated in Figure 1C,D, C0 PSCA+ tumour cells were the most numerous and distributed in all tissue types. C5 MUC2+ tumour cells were the least numerous and originated from all tissue types except metastatic tissues, but only accounted for 0.1% in diffuse tissues, indicating that they may not be the product of metastatic cancers. In contrast, a mere 0.2% of C2 UBE2C+ tumour cells were observed in the G1 phase, while the majority were predominantly identified in the G2M and S phases. This observation implies a potential intricate association between C2 UBE2C+ tumour cells and the processes of mitosis and genetic material replication within GC cells, with DNA replication being one of the fundamental pillars supporting cellular proliferation. Bubble plot showcased the differential expression of marker genes across distinct cell types, as well as the expression of these marker genes in diverse tissue contexts (Figure 1E). The UMAP plots, on the other hand, efficiently elucidated the distribution of marker genes within each category of tumour cells (Figure 1F). Simultaneously, to enhance our understanding of the diverse characteristics in distinct subsets of GC cells, we performed an analysis highlighting the distribution and expression level of CNVscore, nCount_RNA, S.score and G2M.score across various tumour cell subgroups (Figure 1G,H). Consistent with the aforementioned discoveries, tumour cells denoted as C2 UBE2C+ exhibited the most elevated S.score and G2M.score, along with heightened nCount_RNA levels, suggesting a heightened state of cellular cycle activity and proliferation. Conversely, tumour cells designated as C1 CLDN7+ displayed the highest CNVscore.

### 3.3 Biological function analysis of C2 UBE2C+ tumour cells

**FIGURE 2:**
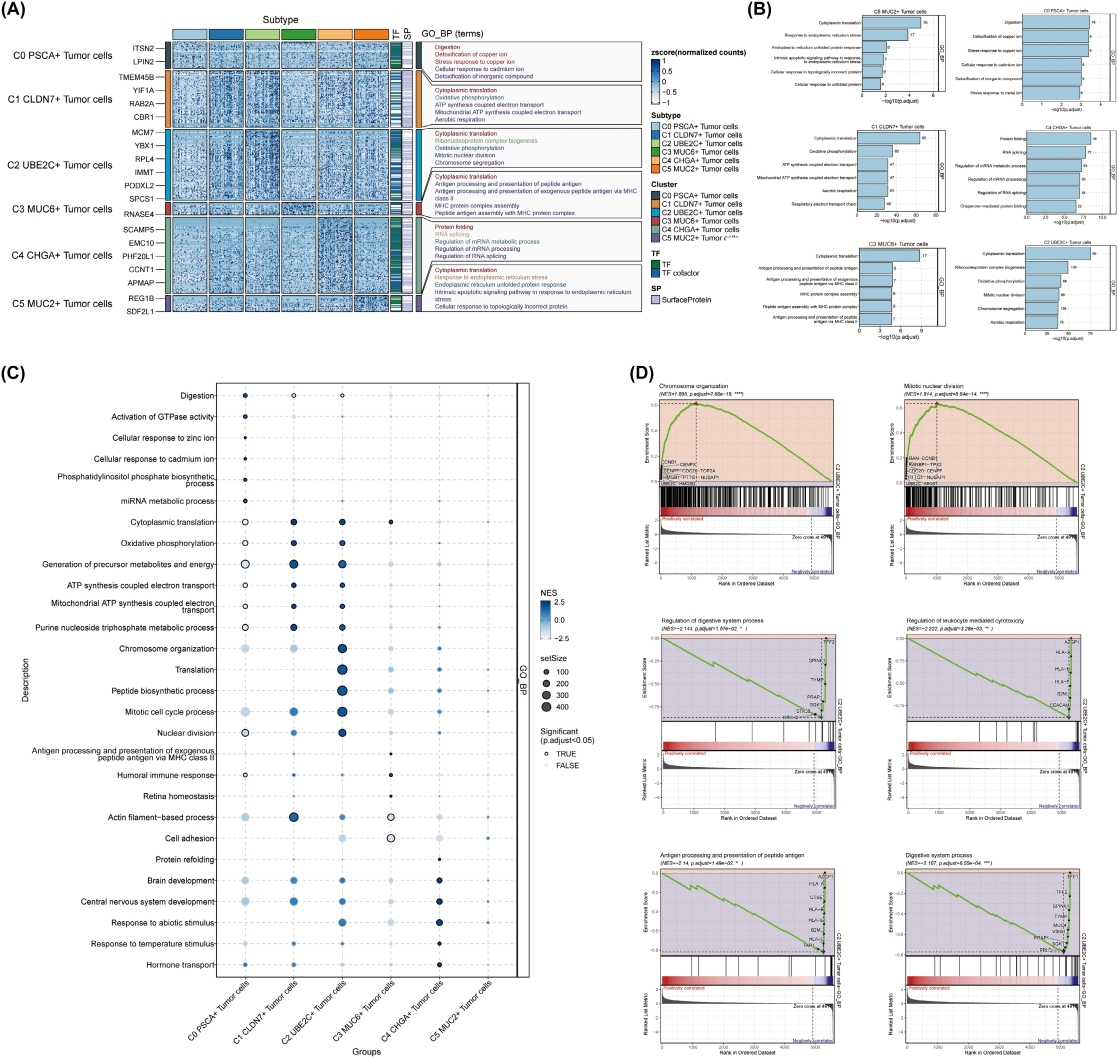
Enrichment analysis of biological functions of tumour cells in GC. (A) Heatmap showing top 5 enrichment entries of GO‐BP enrichment analysis of differential genes in different tumour cell types. (B) Bar plots showing GO‐BP enrichment analysis of differentially expressed genes in different tumour cell types. (C) Bubble plot showing the results of GSEA performed on different tumour cell type differential genes. The bubble size indicates the number of genes enriched in the entry and the colour represents the NES score. (D) GSEA results of pathways associated with differential genes in C2 UBE2C+ tumour cells.

We analysed the functional differences of tumour cells in order to attain a more comprehensive comprehension of the biological attributes exhibited by diverse tumour cell subtypes, while simultaneously elucidating the underlying molecular mechanisms governing the growth of C2 UBE2C+ tumour cells. Based on the outcomes derived from the GO_BP enrichment analysis, it became apparent that C2 UBE2C+ tumour cells were predominantly enriched in pathways such as ‘Cytoplasmic translation’, ‘Ribonucleoprotein complex biogenesis’, ‘Mitotic nuclear division’ and ‘Chromosome segregation’ (Figure 2A,B). These findings align with the outcomes of our antecedent investigation, which showed that C2 UBE2C+ tumour cells regulate tumour growth and development by affecting the synthesis and replication of tumour DNA. Simultaneously, C2 UBE2C+ tumour cells influence the cells' oxidative phosphorylation pathway. C0 PSCA+ tumour cells, on the other hand, were related to trace elements, which were mainly enriched in the ‘Detoxification of copper ion’, ‘Stress response to copper ion’, ‘Cellular response to cadmium ion’ and ‘Detoxification of inorganic compound’. C1 CLDN7+ tumour cells were mainly enriched in ‘Oxidative phosphorylation’, ‘ATP synthesis coupled electron transport’, ‘Mitochondrial ATP synthesis coupled electron transport’ and ‘Aerobic respiration’, which are closely related to cellular energy metabolism. Such findings insinuate an intertwined relationship between C1 CLDN7+ tumour cells and perturbations in ATP homeostasis, potentially expediting the creation of a tumour cell‐supportive microenvironment. The involvement of C3 MUC6+ tumour cells in the MHC molecule‐mediated pathways, pertaining to peptide antigen presentation, assumes paramount significance. These pathways encompassed ‘Antigen processing and presentation of peptide antigen’, ‘Antigen processing and presentation of exogenous peptide antigen via MHC class II’, ‘MHC protein complex assembly’ and ‘Peptide antigen assembly with MHC protein complex’. It is plausible that these pathways bear relevance to the phenomenon of immune evasion exhibited by tumours. The pathways of ‘Protein folding’, ‘RNA splicing’, ‘Regulation of mRNA metabolic process’, ‘Regulation of mRNA processing’ and ‘Regulation of RNA splicing’ exhibited heightened enrichment in C4 CHGA+ tumour cells. These pathways may exert a profound influence on the processing of RNA. Endoplasm reticulum‐associated pathways, encompassing ‘Response to endoplasmic reticulum stress’, ‘Endoplasmic reticulum unfolded protein response’ and ‘Intrinsic apoptotic signalling pathway in response to endoplasmic reticulum stress’, exhibited a predominant focus within C5 MUC2+ tumour cells. In the TME, sustained endoplasmic reticulum stress may engender immunological disruption. In addition, we conducted a GSEA analysis to gain a profound comprehension of the functional attributes specific in C2 UBE2C+ tumour cells. Figure 2C visually depicted the remarkable enrichment of C2 UBE2C+ tumour cells in the realms of ‘Chromosome organization’, ‘Translation’ and ‘Mitotic cell cycle process’. Further GSEA enrichment analysis of C2 UBE2C+ tumour cells showed that C2 UBE2C+ tumour cells revealed positive enrichment in ‘Chromosome organization’ and ‘Mitotic nuclear division’ and negative enrichment in ‘Regulation of digestive system process’, ‘Regulation of leukocyte mediated cytotoxicity’, ‘Antigen processing and presentation of peptide antigen’ and ‘Digestive system process’ (Figure 2D).

### 3.4 Differentiation characteristics of C2 UBE2C+ tumour cells

**FIGURE 3:**
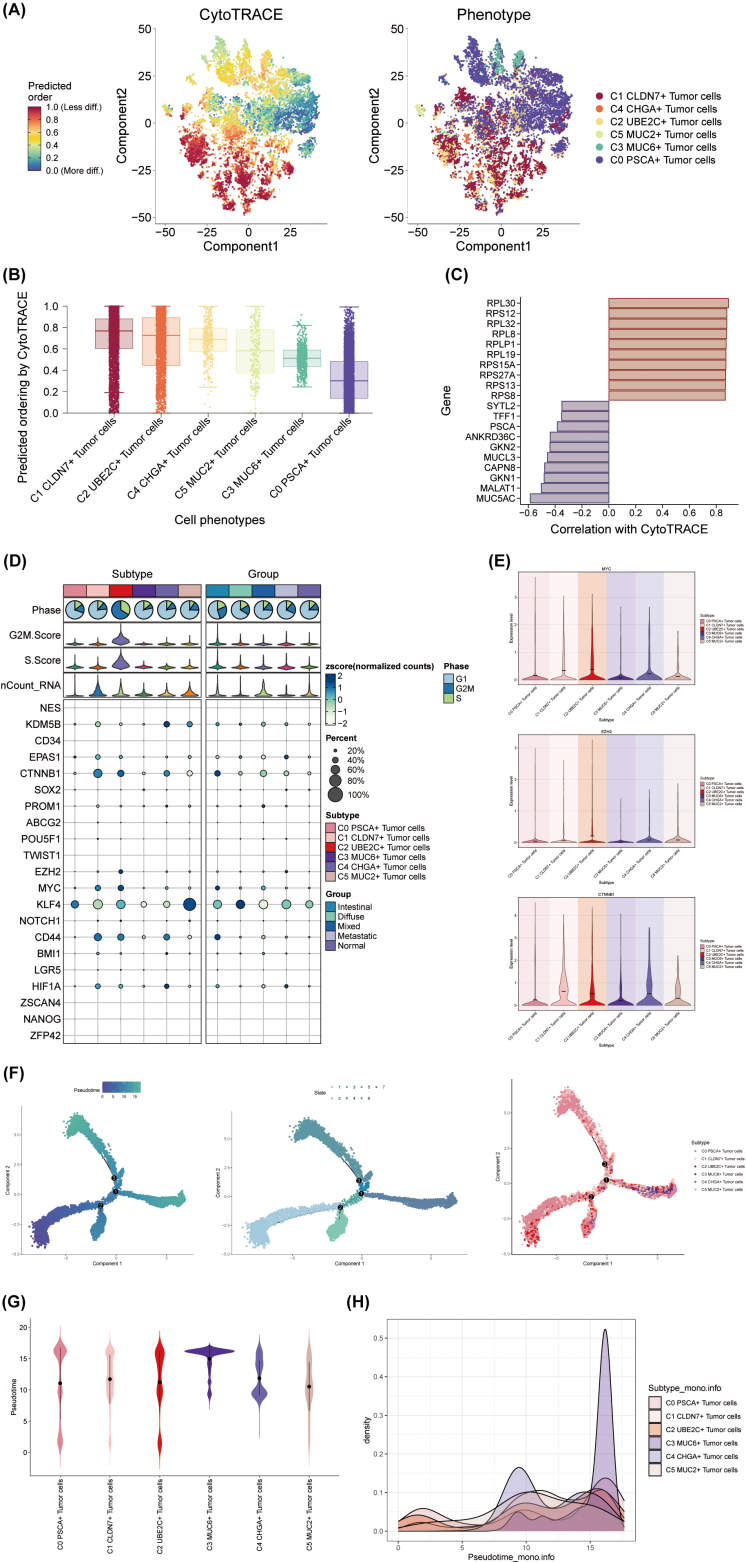
CytoTRACE and Monocle pseudotime analysis of GC tumour cell subpopulations. (A) The left panel demonstrating the distribution of predicted order speculated by CytoTRACE in tumour cells. The colour represents high or low cell stemness. The right panel showing the distribution of tumour cell types. The colour represents different cell subpopulations. (B) The boxplot that ranks the stemness of tumour cell subsets according to CytoTRACE. (C) Bar plot demonstrating genes associated with driving tumour cell stemness. (D) Bubble plot demonstrating differential expression of stemness genes in different tumour cell types and their expression in different tissue types. Bubble sizes indicate the percentage of gene expression and colours are based on normalized data. (E) Violin plots demonstrating the expression levels of stemness genes EZH2, MYC and CTNNB1 in different tumour cell types. (F) Pseudotemporal trajectory plots showing Monocle‐predicted differentiation trajectories of tumour cells. The distribution of pseudotime order (left), state (middle) and cell subtype (right) by Monocle are shown, respectively. (G) Violin plot demonstrating the pseudotime distribution of different tumour cell subtypes. (H) Ridgeline plot showing the density of different types of tumour cells at different times.

**FIGURE 4:**
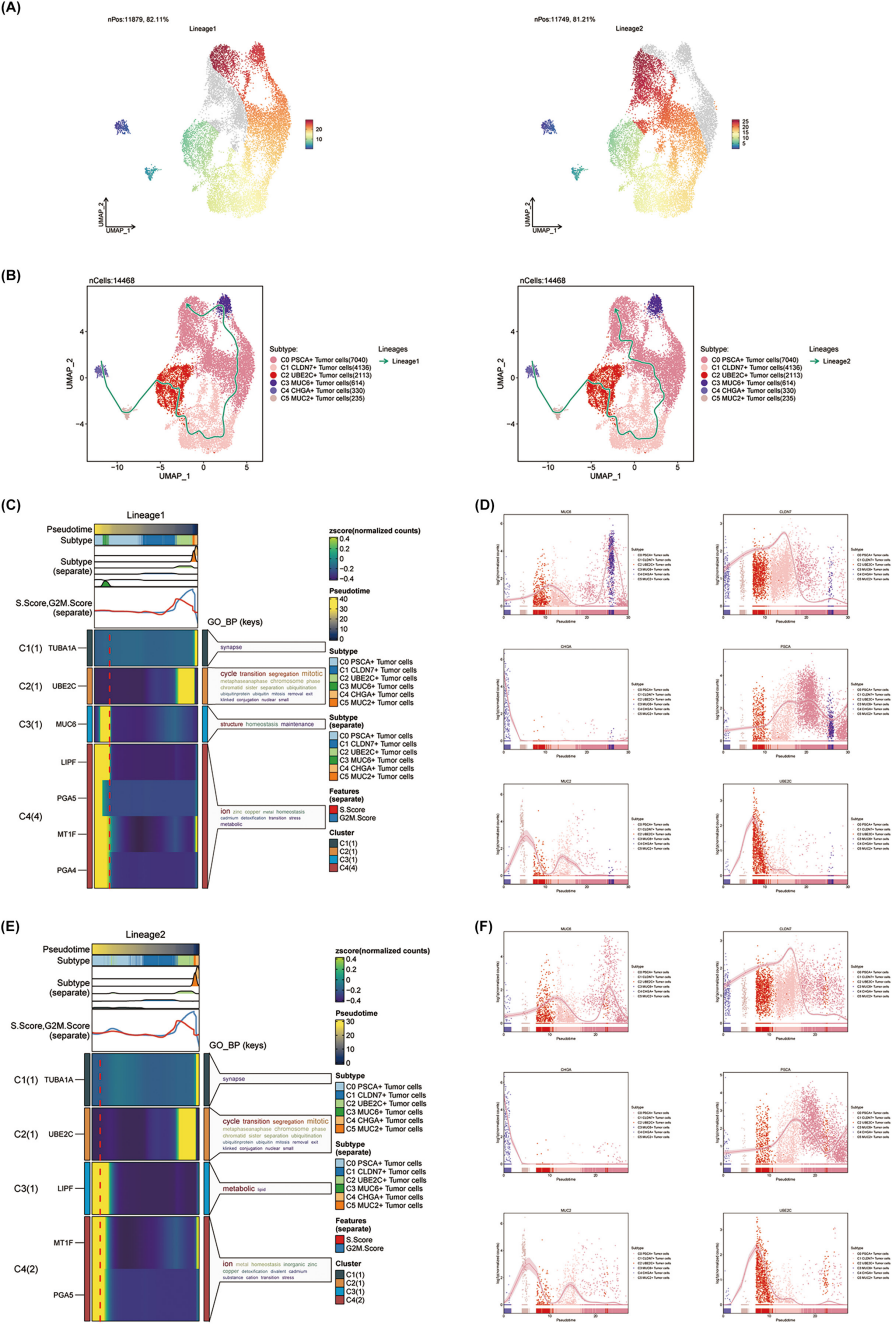
Slingshot analysis of GC tumour cell subpopulations. (A) Demonstration of the distribution of slingshot‐predicted tumour cell differentiation trajectories among all tumour cells by UMAP plots. (Plotting each spectrum according to the pseudotime value to infer the differentiation trajectory, the colour from blue to red indicates the pseudotime from naïve to mature, and the grey part of the cells represent not belonging to the lineage.) Lineage1 nPos: 11,879, 82.11%, lineage2 nPos: 11,749, 81.21%. (B) UMAP plots of the distribution of all tumour cell types in 2 differentiation trajectories. Solid lines indicate differentiation trajectories and arrows point to the direction of differentiation (from naïve to mature). Lineage1: C4 → C5 → C2 → C1 → C0 → C3 → C0; lineage2: C4 → C5 → C2 → C1 → C0. (C, D) Heatmap (left) showing the related characteristics of pseudotime trajectory lineage1 of tumour cells: pseudotime, subtype, features, cluster, GO_BP (keys). The kinetic trend plots (right) demonstrating the dynamic expression of maker gene of different tumour cell type in pseudotime in lineage1 track, colour‐coded by cell type. (E, F) Heatmap (left) demonstrating the related characteristics of pseudotime trajectory lineage2 of tumour cells: pseudotime, subtype, features, cluster, GO_BP (keys). The kinetic trend plots (right) demonstrating the dynamic expression of maker gene of different tumour cell type in pseudotime in lineage2 track, colour‐coded by cell type.

We employed the CytoTRACE methodology to prognosticate and assign scores to cellular stemness in diverse cells, thereby evaluating the differentiation and developmental relationships between different tumour cell subpopulations (Figure 3A,B). Evidently, the development of cell subpopulations followed a sequential pattern of C1‐C2‐C4‐C5‐C3‐C0, as elegantly depicted in Figure 4B, with the C2 UBE2C+ tumour cells positioned at the early stage of tumour differentiation. Subsequently, we undertook gene mining to identify genes associated with the stemness of tumour cells, employing the CytoTRACE methodology. The findings illuminated a robust correlation between ribosome‐encoded genes, such as RPL30, RPS12, RPL32, RPL8 and RPLP1, and cell differentiation processes (Figure 3C). And there is an intricate interplay between ribosomal RNA methylation and the transformative potential as well as self‐renewal capacity of tumour stem cells^13^ In order to delve deeper into the intricate interplay between genes associated with tumour cell stemness and the process of cell differentiation, we employed bubble plot to depict the differential expression of stemness genes across 6 distinct tumour cell types (Figure 3D). MYC, EZH2 and CTNNB1 were the primary stemness genes with high expression levels in C2 UBE2C+ tumour cells (Figure 3E).

Subsequently, we employed the Monocle software to embark on a profound exploration of the intricate origins and progression of tumour cells. This study speculated a potential pseudotemporal trajectory for us, unveiling the process of tumour cell origin and gradual differentiation. The pseudotemporal trajectory of tumour cells, as depicted in Figure 3F, started from the lower left quadrant, and throughout the successive differentiation, it generated three bifurcations, each branch unfolding along a different trajectory. Consistent with the findings from CytoTRACE analysis, the C2 UBE2C+ tumour cells were significantly concentrated in the initial stage of tumour cell development. Meanwhile, we observed the pseudotemporal distribution of different types of tumour cells by violin plot and ridgeline plot (Figure 3G,H).

### 3.5 Slingshot analysis reveals two differentiation trajectories of GC tumour cells

Using Slingshot trajectory analysis, we also investigated the pseudotime trajectory and differentiation trajectories of tumour cell subpopulations. Two tumour cell differentiation trajectories predicted by Slingshot are displayed (Figure 4A,B). In harmony with the aforementioned findings, C2 UBE2C+ tumour cells were still at an earlier stage of tumour cell differentiation, with higher cell proliferation activity, faster tumour growth and greater susceptibility to disease progression and metastasis. Subsequently, we scrutinized the biological functions connected to these two differentiation trajectories by GO_BP analysis, elegantly depicted in Figure 4C,E. The findings demonstrated that C2 UBE2C+ tumour cells were primarily enriched in ‘cycle’, ‘transition’, ‘segregation’ and ‘mitotic’ biological processes in both trajectories. These findings further implicate the importance of C2 UBE2C+ tumour cells in cell differentiation and cell cycle, which may have profound implications on the progression of malignant tumours. Lastly, we used kinetic trend plots to examine the marker gene distribution in different tumour cell subsets on lineage1 and lineage2 throughout pseudotime (Figure 4D,F). The expression of the marker genes MUC6 and MUC2 undergoes rapid upregulation at the forefront of both lineages, followed by a subsequent rapid downregulation as the lineages progress. However, during the early stages of lineages, the expression of the marker gene UBE2C remained in a state of rapid upregulation. This stage likely witnessed a robust proliferation and division of a substantial number of tumour cells. The expression of CLDN7 and PSCA exhibited a gradual increase from the early developmental stage and only accelerated once the expression of UBE2C diminished. In summary, the expression patterns of these six marker genes along the developmental trajectory aligned with their respective defined cell subtypes.

### 3.6 The potential communication network between C2 UBE2C+ tumour cells and other cells

**FIGURE 5:**
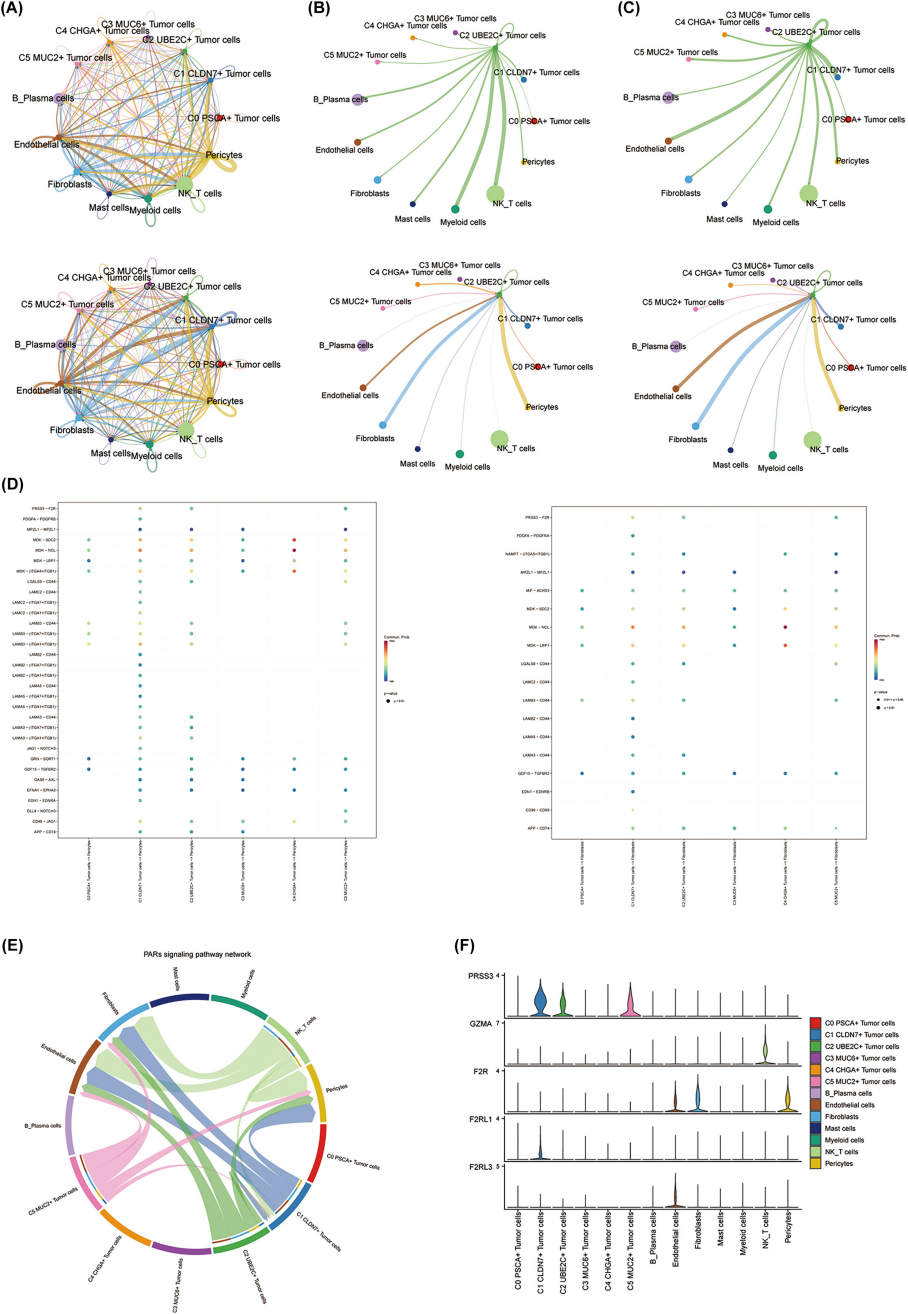
Cell–cell crosstalk patterns in the GC immune microenvironment. (A) Circle plots demonstrating the strength (top) and number (bottom) of all cell–cell interactions. (B) Circle plots of cell–cell interactions intensity for C2 UBE2C+ tumour cells as source (top) and target (bottom). (C) Circle plots demonstrating the number of cell–cell interactions of C2 UBE2C+ tumour cells as source (top) and target (bottom). (D) Bubble plots demonstrating the cellular communication network between tumour cells and pericytes (left) and fibroblasts (right) (screening tumour cells as signal senders and pericytes (left) and fibroblasts (right) as signal receivers). (E) Circle plot showing the intercellular communication network of PARs signalling pathway in tumours. Line widths indicate communication probabilities. (F) Violin plot showing the expression of signalling genes involved in the inferred PARs signalling network.

We employed CellChat for the exploration of cellular communication, aiming to deduce the reciprocal interplay amid C2 UBE2C+ tumour cells and other cells, as depicted in Figure 5A. Among these, in the circle plots with C2 UBE2C+ tumour cells as the target, the communication signals between pericytes, fibroblasts and C2 UBE2C+ tumour cells were stronger (Figure 5B,C). Based on this, we then unveiled the intricate cellular communication network existing amidst tumour cells with pericytes and fibroblasts through the utilization of bubble plots (Figure 5D). Pericytes, owing to their close association with stem cells, possess a myriad of differentiation potentials that play a pivotal role in orchestrating vascular neogenesis, promoting vascular maturation, as well as facilitating tissue repair and regeneration.^14^ Fibroblasts possess remarkable reversibility, enabling them to re‐enter the cell cycle and thereby exert influence over cellular phenotypes and the progression of disease processes. Moreover, fibroblasts exhibit high plasticity, actively contributing to tissue repair endeavours.^15^ Both cells play unique roles in cell differentiation and cell cycle and are closely related to C2 UBE2C+ tumour cells. Protease‐activated receptors (PARs), as members of the G protein‐coupled receptor family, possess the ability to orchestrate the activation of the Extracellular Signal‐Regulated Kinase (ERK) signalling transduction pathway. It is worth noting that the sustained activation of ERK1/2 plays an indispensable role in facilitating the progression from the G1 phase to the S phase.^16^ PARs are, therefore, essential for the cell cycle transition. To explore this phenomenon, we scrutinized the intercellular communication network based on the PARs signalling pathway and investigated the expression of signalling genes implicated in the PARs signalling network (Figure 5E,F). Notably, our findings revealed that C2 UBE2C+ tumour cells had stronger efferent signals in the PARs signalling pathway, and actively interacted with pericytes, fibroblasts and endothelial cells, and high expression levels of the involved signalling gene PRSS3.

### 3.7 Knockdown of CREB3 impedes the proliferation, migration and invasion of GC cells

**FIGURE 6:**
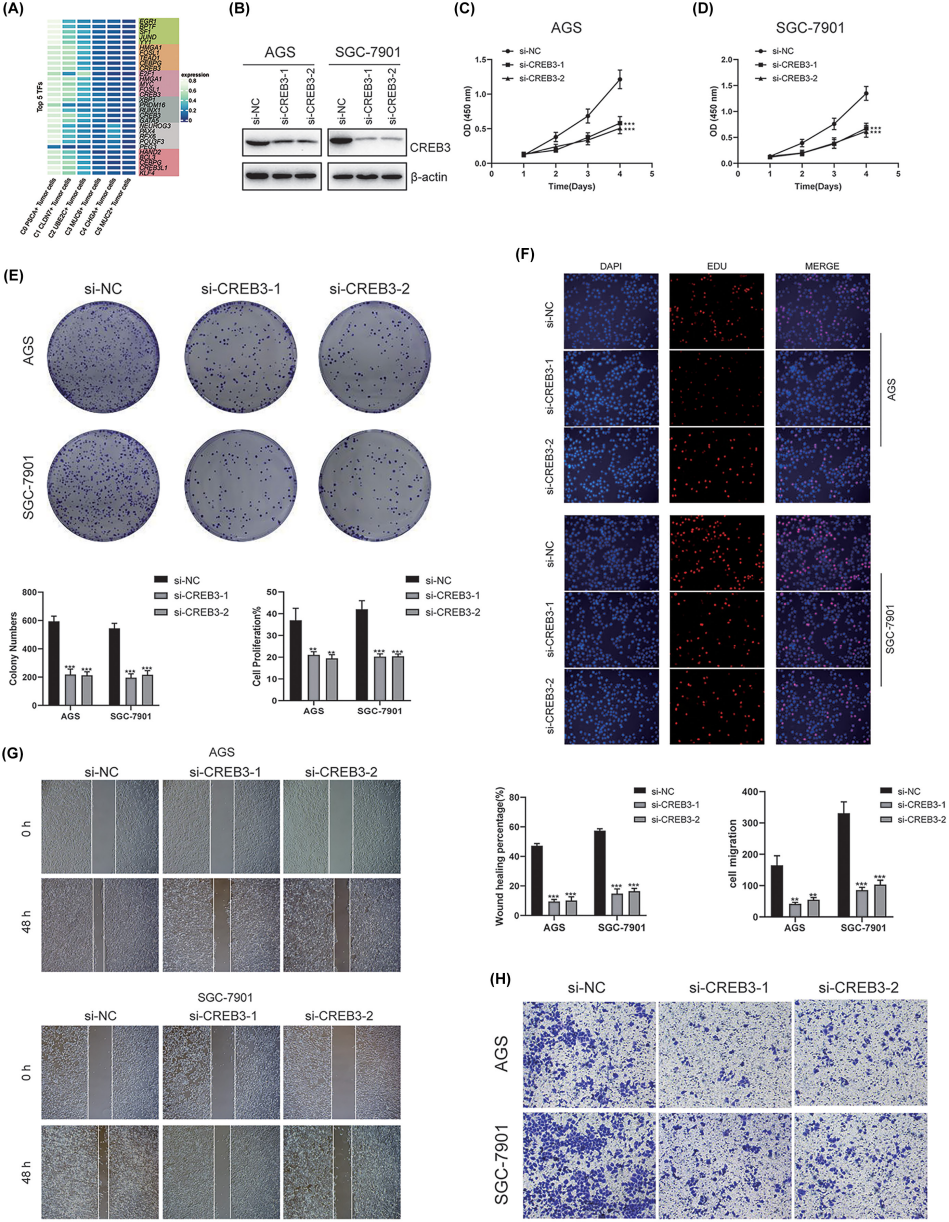
In vitro experimental validation of CREB3. (A) Heatmap demonstrating top 5 TFs in different tumour cell types. (B) Western blot detects protein expression levels after CREB3 knockdown. (C, D) CCK‐8 assay showed a significant decrease in cell viability after CREB3 knockdown. (E) The colony formation experiment showed that the number of colonies of CREB3 knockdown cells was significantly lower than that of si‐NC group. (F) EdU staining assay showed that CREB3 knockdown hindered the proliferation of AGS and SGC‐7901 cells. (G) The scratch experiments showed that CREB3 knockdown significantly slowed down the migration of AGS and SGC‐7901 cells. (H) Transwell experiments showed that CREB3 knockdown significantly slowed down the invasion of AGS and SGC‐7901 cells. **p < 0.01, ***p < 0.001.

In order to delve deeper into the gene regulatory network associated with C2 UBE2C+ tumour cells, we employed pySCENIC to assess the specific regulation of diverse tumour cell populations (Figure 6A). Our findings revealed that the TFs E2F1, HMGA1, MYC, FOSL1 and CREB3 exhibited exceptional levels of activity within C2 UBE2C+ tumour cells. CREB3 is located on the endoplasmic reticulum membrane and contains five family members (CREB3 and its homologues CREB3L1, CREB3L2, CREB3L3 and CREB3L4), which primarily exert influence on cellular metabolic processes.^17^ Aberrant expression of CREB3 has been observed in diverse cancer types such as breast and bladder cancers, which can affect tumour progression and metastasis.^18^ Nonetheless, the precise involvement of CREB3 in GC remains to be elucidated.

Consequently, we performed in vitro functional experiments to validate the influence of CREB3 on GC cells. First, following CREB3 knockdown in both cell lines, Figure 6B demonstrated a considerable reduction in the protein level of CREB3. Moreover, the CCK‐8 assay exhibited a notable decrease in tumour cell viability within the CREB3 knockdown group when compared to the control group (Figure 6C,D). Simultaneously, the colony formation experiment and EdU staining assay further substantiated that, upon the knockdown of CREB3, cellular proliferation was significantly attenuated in both cell line models (Figure 6E,F). These findings indicate that the knockdown of CREB3 hinders tumour cell activity and proliferation, consequently impeding tumour growth. Subsequently, we substantiated the influence of CREB3 on the migratory and invasive abilities of tumour cells through scratch and transwell experiments. Consistent with the aforementioned observations, the knockdown of CREB3 also effectively suppressed the migration and invasion of both types of GC cells, yielding statistically significant results (Figure 6G,H). These investigations validate the pivotal significance of the CREB3 in the context of GC tumorigenesis and metastatic potential.

### 3.8 Screening of key genes related to tumour prognosis and construction of UBE2C+ tumour cells score

**FIGURE 7:**
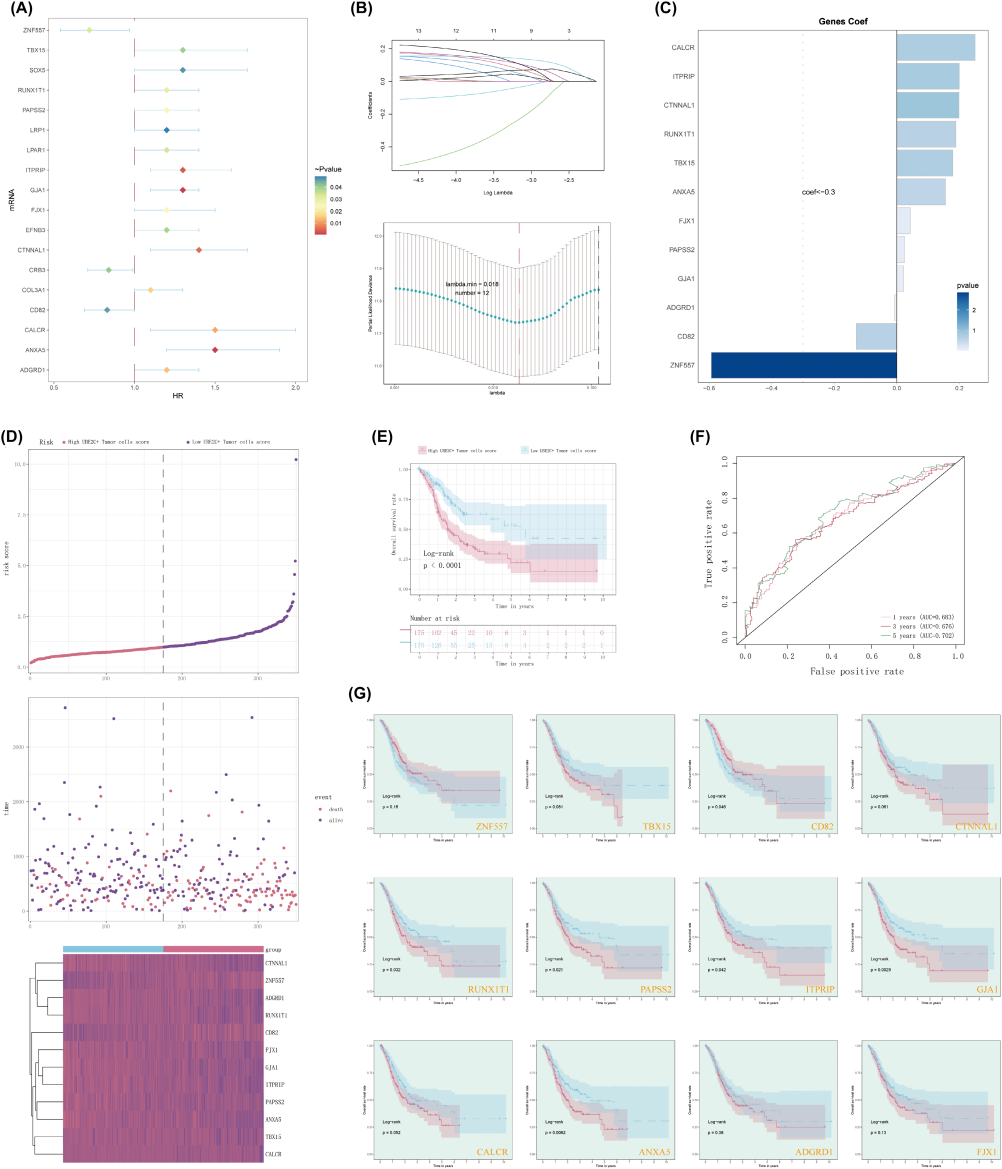
Screening of key genes related to tumour prognosis and construction of UBE2C+ tumour cells score. (A) Univariate Cox regression analysis of prognosis‐related gene risks. (B) LASSO regression analysis modelling of 12 prognosis‐related genes (Ten‐fold cross‐validation of the optimal parameter (lambda) for screening (top). The LASSO coefficient curve is determined by the optimal lambda (bottom)). (C) Coef values for the 12 genes screened by the LASSO Cox regression model. (D) Curve graph showing the risk scores of high UBE2C+ tumour cells score group and low UBE2C+ tumour cells score group (top), scatter plot showing the survival status of the two groups over time survival/death events (middle), heatmap showing the differential expression of UBE2C+ tumour cells score model constituent genes (bottom). (E) Kaplan–Meier curves of survival differences between high UBE2C+ tumour cells score group and low UBE2C+ tumour cells score group. (F) AUC values for predicting outcomes at years 1, 3 and 5 in the TCGA cohort. (G) Survival curves for each gene of the construct model, where ITPRIP, GJA1, RUNX1T1, CD82, PAPSS2 and ANXA5 were more significant (*p* < 0.05).

**FIGURE 8:**
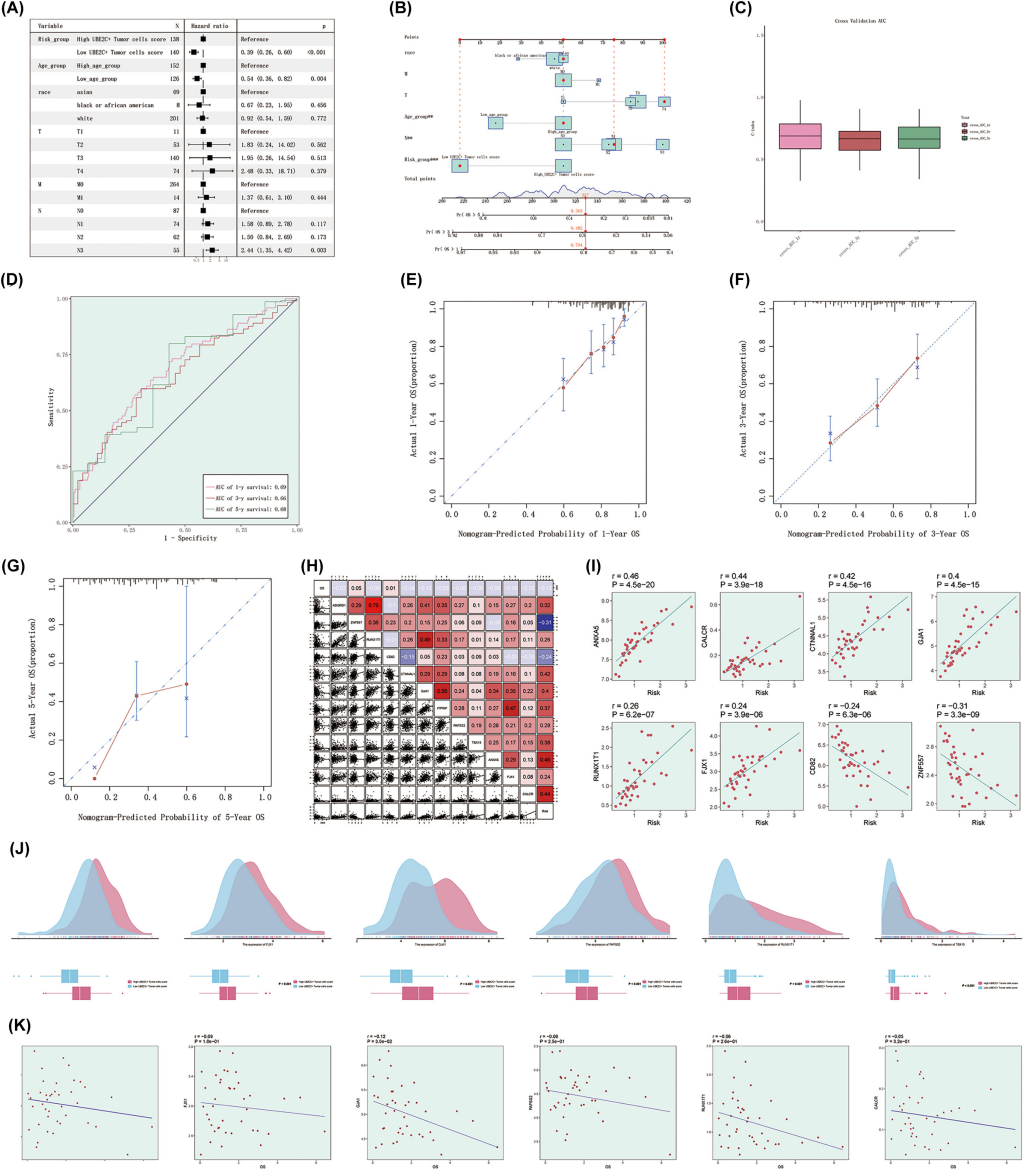
Prognostic model assessment of high UBE2C+ tumour cells score group and low UBE2C+ tumour cells score group based on UBE2C+ tumour cells score constitutive genes. (A) Multivariate Cox analysis forest plot based on UBE2C+ tumour cells score and clinicopathologic factors (age, race, TNM staging). (B) Nomogram showing the predicted 1‐, 3‐ and 5‐year OS of GC patients. (C) C‐index for internal cross‐validation of Nomogram predicted outcomes. (D) AUC values for predicting outcomes at years 1, 3 and 5 in the TCGA cohort. (E–G) Nomogram calibration curves predicting 1‐, 3‐ and 5‐year OS in GC patients. (H) Heatmap demonstrating the correlation between UBE2C+ tumour cells score model constitutive genes, OS and risk scores. (I) Correlation analysis between UBE2C+ tumour cells score model constitutive genes and risk scores. (J) Distribution density and differential expression of UBE2C+ tumour cells score model constitutive genes between high UBE2C+ tumour cells score group and low UBE2C+ tumour cells score group. (K) Correlation analysis between UBE2C+ tumour cells score model constitutive genes and OS.

To elucidate the clinical significance of the UBE2C or CREB3‐associated regulatory network identified in this study, we proceeded to identify the target genes associated with CREB3 and conducted univariate Cox regression analysis on these target genes. Among the 18 genes associated with patient prognosis, ZNF557, CRB3 and CD82 exhibited a protective effect, while the remaining genes were deemed risk factors, as depicted in Figure 7A. In order to avoid multicollinearity of these genes, we further performed LASSO regression analysis, and finally screened to obtain 12 genes that could be utilized to generate a scoring system for UBE2C+ tumour cells following lambda validation (Figure 7B). Among these genes, CD82 and ZNF557 displayed Coef values <0 (Figure 7C), aligning with the findings depicted in Figure 8A and suggesting their significance as protective factors associated with prognosis.

The distribution of the UBE2C+ tumour cell score in the TCGA cohort dataset was illustrated in Figure 7D. Patients in the TCGA cohort were categorized into two groups according to the median UBE2C+ tumour cell score: the high UBE2C+ tumour cell score group and the low UBE2C+ tumour cell score group. Higher scores were associated with a poorer prognosis and an increased number of fatal events. The expression levels of ZNF557 and CD82 exhibited a consistent decline as the score increased, whereas the expression levels of other high‐risk genes gradually rose. Concurrently, survival analysis demonstrated that the group with a low score for UBE2C+ tumour cells had significantly better survival outcomes compared to the group with a high score (Figure 7E). The receiver operating characteristic curves in the TCGA cohort indicated that the AUC values for 1‐, 3‐ and 5‐year OS of GC patients were 0.683, 0.676 and 0.702, respectively (Figure 7F). Furthermore, a survival analysis was conducted on the individual genes comprising the UBE2C+ tumour cell score, and it revealed statistically significant results for ITPRIP, GJA1, RUNX1T1, CD82, PAPSS2 and ANXA5 (Figure 7G).

### 3.9 Evaluation of prognostic model based on UBE2C+ tumour cell score

To further validate the effectiveness of UBE2C+ tumour cells score as an autonomous prognostic indicator for GC prognosis, we constructed a clinical predictive model. Figure 8A illustrated the outcomes of multivariate Cox analysis incorporating the UBE2C+ tumour cell score, age, race and TNM stage, thereby indicating that the UBE2C+ tumour cell score holds independent prognostic significance for GC patients (*p* < 0.05). Nomogram based on age, race and TNM stage validated the good predictive ability of risk scores for 1‐, 3‐ and 5‐year OS in GC patients (Figure 8B,C). Meanwhile, we also forecasted the AUC values of 1‐, 3‐ and 5‐year OS of GC patients again in the TCGA cohort, and the findings demonstrated that the AUC values were all greater than 0.6 (Figure 8D). Excellent clinical usefulness of the UBE2C+ tumour cell score model was also confirmed by the substantial overlap of the calibration curves between the actual 1‐, 3‐ and 5‐year survival rates and the survival rates predicted by nomogram (Figure 8E–G). These findings underscore the robust predictive capability of the UBE2C+ tumour cell score model in determining the survival outcomes of GC patients.

Following the validation of the UBE2C+ tumour cell score model in predicting the prognosis of patients with GC, we proceeded to investigate the association between the modelled genes, risk scores and OS (Figure 8H). The findings endorsing the protective nature of ZNF557 and CD82 variables are reinforced by the inverse correlation observed between their expression levels and risk scores, as depicted in Figure 8I. Furthermore, our focus extended to examining the correlation between risk genes and OS, along with the disparities in their expression between the high UBE2C+ tumour cell score group and the low UBE2C+ tumour cell score group (Figure 8J,K). Undoubtedly, an inverse correlation existed between risk genes and OS.

### 3.10 Differential analysis of immune infiltration

Subsequently, we scrutinized the disparities in immune cell infiltration among the cohorts characterized by high and low UBE2C+ tumour cell scores. Employing the CIBERSORT methodology, we initially evaluated the allocation of immune cells between the groups exhibiting high and low UBE2C+ tumour cell scores, as illustrated in Figure S3A. Moreover, we conducted an additional analysis to assess the estimated proportion of each immune cell types in GC (Figure S3B). Notably, the estimated proportion of T cells CD4 memory resting was the highest, closely followed by Macrophages M0 and Macrophages M2. Furthermore, the proportion of T cells CD8 also demonstrated a comparatively substantial presence. Subsequently, we proceeded to compare the disparities in immune cell infiltration between the groups (Figure S3C). In contrast to the high UBE2C+ tumour cells score group, the low UBE2C+ tumour cells score group exhibited a higher proportion of B cells memory and T cells CD4 memory activated, and a lower proportion of Macrophages M2 and Monocytes, suggesting that the low UBE2C+ tumour cells score group may have better anti‐tumour immune effects and less immunosuppression. To gain deeper insights into the contrasting patterns of immune infiltration among these two groups, we proceeded with a detailed assessment of the correlation between immune cells and the UBE2C+ tumour cells score. Among these cell types, macrophages M2, neutrophils, mast cells activated, and eosinophils displayed a positive correlation with the risk scores. In contrast, T cells CD4 memory activated, B cells memory and T cells regulatory (Tregs) exhibited a negative correlation with the risk scores (Figure S3D). Consequently, we postulated the presence of a potentially more favourable immune microenvironment within the low UBE2C+ tumour cell score group.

Subsequently, we examined the correlation between immune cells and UBE2C+ tumour cells score model constitutive genes, OS and risk scores (Figure S3E). It is noteworthy that upon employing the ESTIMATE algorithm to calculate the disparities in stromal score, immune score, ESTIMATE score and tumour purity between the two groups, we observed significantly lower stromal and ESTIMATE scores in the low UBE2C+ tumour cell score group compared to the high group. Furthermore, the tumour purity in the low UBE2C+ tumour cell score group was higher than that in the high UBE2C+ tumour cell score group. However, no substantial difference in immune score existed between the two groups (Figure S3F,G). To provide a more visually comprehensive representation of the immune microenvironment pattern under varying UBE2C + tumour cell score conditions, we employed a heat map to depict the gene expression, stromal score, immune score, ESTIMATE score, tumour purity and the degree of immune cell infiltration between the two group (Figure S3H). Finally, we assessed the correlation between immune checkpoint‐associated genes, UBE2C+ tumour cells score composition genes, OS and risk scores, as well as immune checkpoint‐associated gene expression level differences between the two groups (Figure S3I,J). Among them, there is a significant expression level difference between the two groups and a strong negative correlation between LGALS9, TNFRSF14 and risk scores. These findings imply that LGALS9 and TNFRSF14 may offer guidance for the future development of GC immune checkpoint inhibitors.

### 3.11 Enrichment analysis of high UBE2C+ tumour cells score group and low UBE2C+ tumour cells score group

In order to ascertain the disparities in biological functions between the high UBE2C+ tumour cell score group and the low UBE2C+ tumour cell score group, we conducted an enrichment analysis. The outcome of the volcano plot indicated a prominent distinction in gene expression between the two groups (Figure S4A). The genes displaying differential expression between the two groups were subsequently subjected to enrichment analysis. Initially, GO analysis was conducted, revealing several pathways associated with the differentially expressed genes. These pathways included ‘keratinization’, ‘keratinocyte differentiation’, ‘epidermal cell differentiation’, ‘epidermis development’ and ‘skin development’(Figure S4B). KEGG analysis showed that differential genes were significantly enriched in the ‘PPAR signalling pathway’, ‘Neuroactive ligand‐receptor interaction’, ‘Cholinergic synapse’, ‘Protein digestion and absorption’ and ‘Focal adhesion’ pathways (Figure S4C). Additionally, Figure S4D presents the findings from the GSEA analysis.

### 3.12 Mutation analysis of high UBE2C+ tumour cells score group and low UBE2C+ tumour cells score group

To comprehend the mutated genes within the tumours, we conducted an analysis of the mutations present in cells belonging to both the high UBE2C+ tumour cell score group and the low UBE2C+ tumour cell score group. Figure S5A displays the top 20 mutated genes, with the top bar indicating the mutation load per sample and the right bar indicating the total percentage of mutations in this gene in all samples, with TTN having the highest mutation frequency. The heatmap demonstrated the mutation correlation analysis between the constitutive genes of the UBE2C+ tumour cells scoring model (Figure S5B), and the lollipop chart visually represents the mutation spectrum of select genes within this model (Figure S5C). Subsequently, we explored the disparity in TMB between the high UBE2C+ tumour cell score group and the low UBE2C+ tumour cell score group. Notably, the low UBE2C+ tumour cell score group exhibited a significantly elevated TMB compared to the high UBE2C+ tumour cell score group, with a statistically significant distinction (*p* < 0.01) between the two cohorts (Figure S5D). Furthermore, a negative correlation between TMB and the risk scores was observed (Figure S5E). We categorized the tumours into high TMB and low TMB groups based on our TMB scores. The OS rate of high TMB groups was higher than that of low TMB groups, which is in line with the results above (Figure S5F). To further elucidate the association between TMB, UBE2C+ tumour cell score and OS, we integrated the TMB score and UBE2C+ tumour cell scores to classify patients into four distinct groups: high UBE2C+ tumour cells score high TMB score, high UBE2C+ tumour cells score low TMB score, low UBE2C+ tumour cells score high TMB score and low UBE2C+ tumour cells score low TMB score. As shown in Figure S5G, the OS rate was highest in the low UBE2C+ tumour cells score high TMB score group, followed low UBE2C+ tumour cells score low TMB score group, while there was little difference in OS rate between the high UBE2C+ tumour cells score high TMB score group and the high UBE2C+ tumour cells score low TMB score group. This reaffirms the previous findings. Lastly, we conducted a CNV analysis on the constitutive genes of the UBE2C+ tumour cell score (Figure S5H). The analysis revealed that although CNV events were observed in the target genes, their occurrence was infrequent. Notably, PAPSS2 exhibited the highest frequency of CNV loss, whereas CALCR showed the highest frequency of CNV gain.

### 3.13 Drug sensitivity analysis

Finally, we conducted a detailed examination of the variations in drug responsiveness among the cohorts characterized by high and low UBE2C+ tumour cell scores (Figure S5I). The results revealed discernible disparities in medication sensitivity between the groups exhibiting high and low UBE2C+ tumour cell scores. For instance, Docetaxel, a frequently utilized chemotherapeutic agent in the clinical setting for the treatment of GC, exhibited heightened sensitivity in the group characterized by a high UBE2C+ tumour cell score. Conversely, AKT inhibitor VIII, an AKT inhibitor that responds well in GC, displayed enhanced sensitivity in the cohort characterized by a low UBE2C+ tumour cell score.^19^

### 3.14 Workflow

The flow chart for this study is shown in Figure S6.

## 4 DISCUSSION

In this study, we first demonstrated the GC microenvironment landscape using scRNA‐seq. Consistent with previous studies,^20^, ^21^, ^22^ we ascertained a diverse array of cell subsets within GC, encompassing NK_T cells, B_Plasma cells, epithelial cells and fibroblasts. Furthermore, we scrutinized the sample source of these cells and their distribution across the cell cycle. Remarkably, an abundance of proliferative immune infiltrating cells, notably NK_T cells and B_Plasma cells, were observed within the tumour tissue. Previously, it was postulated that immune cells play a pivotal role in immune surveillance, capable of hindering tumour growth by instigating immune responses.^23^ However, contemporary investigations into the tumour immune microenvironment have substantiated that certain immune cells have undergone a loss of their anti‐tumour immune efficacy, and in fact, contribute to fostering an immunosuppressive microenvironment supportive of tumour cell survival. These cells assume a collaborative role in propelling tumour progression. Consequently, even though immune checkpoint inhibitors (ICIs) have been used in the treatment of refractory GC and improve the OS rate to some extent, the curative effect is still not completely satisfactory.^1^, ^24^, ^25^

Alongside immunotherapy, chemotherapy continues to prevail as the primary approach for treating GC. Certain chemotherapeutic agents exhibit targeted interactions with particular cell cycles, exemplified by antipyrimidine medications like 5‐Fu and taxanes such as docetaxel. Nevertheless, it should be acknowledged that these drugs lack tumour specificity, as they can impact the cell cycles of normal cells as well, consequently resulting in the rapid development of drug resistance.^26^ Similarly, the targeted drug trastuzumab has demonstrated favourable therapeutic efficacy solely among patients exhibiting Her2 mutation.^27^, ^28^ Currently, there exists an imperative necessity to discover more potent therapeutic interventions, in conjunction with chemotherapy or alternative modalities, to more effectively impede tumour proliferation and protract the overall survival period for individuals afflicted with GC.

Given the paramount influence of cancer cells on the TME, our research concentrates specifically on GC cells. It is notable that we have identified a distinct cell population within tumours, characterized by a notable upregulation of UBE2C. This cell population is predominantly localized within the G2M and S phases, while representing a mere 0.2% of the G1 phase. Remarkably, such findings have not been previously reported in scientific literature. Additional validation provided by the S.score and G2M.score reinforces the importance of these findings. These observations strongly imply a close association between UBE2C+ tumour cells and the rapid proliferation and progression of malignancies.

The protein ubiquitination serves as a dynamic and intricate post‐translational modification, playing a pivotal role in the targeted degradation of proteins within cellular systems. This process holds significant influence over a diverse array of biological phenomena, including but not limited to the regulation of the cell cycle, DNA damage response and repair, cellular autophagy, as well as cellular metabolism.^29^, ^30^ The UBE2C, as a coupling enzyme, can participate in cell cycle progression and checkpoint regulation by targeted degradation of short‐lived proteins. UBE2C plays an important role in cyclin B and mitotic spindle checkpoint control and can affect the process of mitotic exit.^31^ Notably, cells exhibiting UBE2C overexpression display a disregard for mitotic spindle checkpoint signals, resulting in compromised genomic stability—a hallmark characteristic of cancer.^32^ Indeed, UBE2C expression is commonly observed to be upregulated in the realm of malignancies and hematologic disorders, displaying a direct association with heightened tumour proliferation and aggressiveness. Our research confirmed these findings in GC. Cell cycle analysis and enrichment analysis showed that C2 UBE2C+ tumour cells had high proliferation and was mainly enriched in DNA synthesis and replication and mitotic pathway. Pseudotime analysis also showed that C2 UBE2C+ tumour cells had higher cell stemness and were in the early stage of cell differentiation and were, therefore, more malignant. More importantly, patients exhibiting high levels of UBE2C expression tend to have a lower survival rate and a higher risk of recurrence compared to those with lower UBE2C expression.^31^, ^33^

Taking into consideration the potential interplay between tumour cells and surrounding cellular components, we conducted an analysis of the cellular communication involving C2 UBE2C+ tumour cells. The findings revealed a robust interaction between UBE2C+ tumour cells and PARs, the regulatory receptors governing the activity of the cell cycle regulatory kinase ERK1/2,^34^, ^35^ thus providing further substantiation for our observations.

The factors contributing to the unfavourable prognosis of GC encompass not only delayed diagnosis and limited treatment options but also the heightened proliferation and formidable invasiveness exhibited by cancerous cells. In our pursuit of identifying genes associated with the prognosis of GC and establishing a risk score model, we once again delved into the exploration of TFs linked to UBE2C. The dysregulation of the CREB3 protein family's expression and control manifests a profound correlation with the onset and progression of tumours and diverse non‐neoplastic ailments. Operating as a pivotal sensor of endoplasmic reticulum stress, CREB3 assumes an indispensable role in the viability of tumour cells when confronted with such stressors. In the case of glioblastoma, heightened expression of CREB3 has been demonstrated to bolster cell proliferation and invasiveness in in vitro studies.^36^ Conversely, in lung cancer, the depletion of CREB3 protein levels have shown to impede tumour progression and induce apoptosis in lung adenocarcinoma cells.^37^ In osteosarcoma, downregulation of CREB3, which is targeted by miR‐203a‐3p, resulted in decreased proliferation and migration of tumour cells, while promoting apoptosis.^38^ These findings have also been corroborated in our functional experiments involving GC cells.

Hence, our endeavour revolves around corroborating whether the remarkably potent transcription factor, CREB3, within UBE2U+ tumour cells, exerts regulatory control over the unrestrained proliferation of GC cells. The findings demonstrate a substantial reduction in the proliferation, invasion and migration of GC cells upon the knockdown of CREB3. Utilizing these findings as a foundation, we conducted differential expression analysis and COX regression analysis on the gene expression data from a cohort of GC patients. Consequently, we identified 12 target genes related to CREB3. Notably, the Single gene survival analysis revealed significant associations between several genes and patients' survival outcomes. Specifically, the gene CD82 exhibited a protective effect, whereas the genes TPRIP, GJA1, Runx1t1, PAPSS2 and ANXA5 entailed risk factors that markedly impacted patient survival. Consonant with our investigation, a multitude of studies have substantiated the inhibitory influence of CD82 on the invasion and migration of GC cells. This effect can likely be attributed to its involvement in modulating the EGFR/ERK1/2‐MMP7 signalling pathway.^39^, ^40^ Furthermore, it has been established that the genes ANXA5 and GJA1, deemed as risk genes, hold significant implications in multiple prognostic models pertaining to GC.^41^, ^42^, ^43^, ^44^ Similarly, the genes PAPSS2 and ITPRIP have been recognized as unfavourable prognostic factors across a spectrum of cancers, including thyroid, colorectal and lung cancers.^45^, ^46^, ^47^ Nevertheless, it is noteworthy to highlight that within the context of GC, the gene RUNX1T1 exhibits a suppressive role, wherein it actively hinders the tumour‐promoting activity of C/EBPβ and restrains tumour proliferation.^48^ Conversely, in the domain of breast cancer, an elevated expression of RUNX1T1 facilitates RBM24‐induced tumour cell proliferation, thereby fostering tumour progression.^49^ RUNXIT1, functioning as a transcriptional co‐repressor, exhibits somatic mutations in numerous solid tumours; however, the precise mutation loci remain largely elusive.^50^ These mutation sites may potentially contribute to divergent tumour outcomes.

The variability in tumour immune infiltration partially accounts for the disparity in prognosis between the two cohorts. Within the low‐risk score group, a greater proportion of immune effector cells and a diminished proportion of immunosuppressive cells were observed, accompanied by heightened tumour purity. These findings imply that tumours within the low‐risk score group exhibit reduced malignancy, heightened immune infiltration and a diminished likelihood of tumour immune evasion. Furthermore, the disparity in TMB constitutes another influential factor impacting patient prognosis. Several investigations have revealed a positive correlation between increased TMB and enhanced survival rates among individuals afflicted with GC.^51^, ^52^ These findings align with our own observations, indicating that patients with higher TMB scores exhibit prolonged survival. Despite the high‐risk group's heightened immunosuppression and inferior prognosis, it is plausible that they may demonstrate heightened sensitivity to the chemotherapeutic agent docetaxel.

## 5 CONCLUSION

In this research endeavour, the application of scRNA‐seq to elucidate the ecological microscope of GC led to the first identification of a unique tumour cell subtype and its associated marker genes, which are significantly interlinked with gastric carcinogenesis and progression. Consequently, the comprehensive analysis of UBE2C+ tumour cells furnish a novel molecular biological standpoint concerning the unbridled proliferation of neoplastic cells. Significantly, our investigation has elucidated the pivotal involvement of the transcription factor CREB3 in the invasion and progression of GC. Furthermore, the risk score model derived from these findings exhibits commendable clinical predictive capability, thereby presenting a fresh perspective for patients' prognosis and therapeutic guidance. Nonetheless, it is essential to acknowledge the limitations associated with this study. Primarily, all our analyses rely exclusively on publicly available database data, and the amalgamation and manipulation of this data may entail the omission of consequential information. Thus, more extensive clinical samples and large‐scale prospective studies are indispensable to corroborate our findings. Secondly, despite conducting in vitro experiments, tumour occurrence and progression occur within a complex in vivo milieu. Consequently, a more comprehensive array of in vivo experiments is requisite to substantiate our findings.

## AUTHOR CONTRIBUTIONS

**Wenjia Zhang:** Data curation (equal); resources (equal). **Xiaojing Wang:** Data curation (equal); software (equal); writing – original draft (equal). **Jiaxing Dong:** Data curation (equal); formal analysis (equal). **Kai Wang:** Investigation (equal). **Wanju Jiang:** Methodology (equal). **Chenchen Fan:** Visualization (equal). **Haitao Liu:** Validation (equal). **Lihong Fan:** Writing – review and editing (equal). **Lei Zhao:** Methodology (equal); writing – review and editing (equal). **Guoshu Li:** Methodology (equal); writing – review and editing (equal).

## CONFLICT OF INTEREST STATEMENT

The authors declare no conflicts of interest.

## Supporting information


Figure S1

Figure S2

Figure S3

Figure S4

Figure S5

Figure S6

Table S1

Table S2

## Data Availability

The datasets of single‐cell sequencing generated and/or analysed during the current study are publicly available in the GEO and TCGA. The original contributions presented in the study are included in the article/Supplementary Material. The inquiries of original contributions presented in the study can be directed to the corresponding authors.
