# Supplementary figures and images for: Single‐cell analysis uncovers high‐proliferative tumour cell subtypes and their interactions in the microenvironment of gastric cancer
Source: J Cell Mol Med. 2024 Jun 19;28(12):e18373. doi: 10.1111/jcmm.18373 (PMC11187953; doi:10.1111/jcmm.18373)

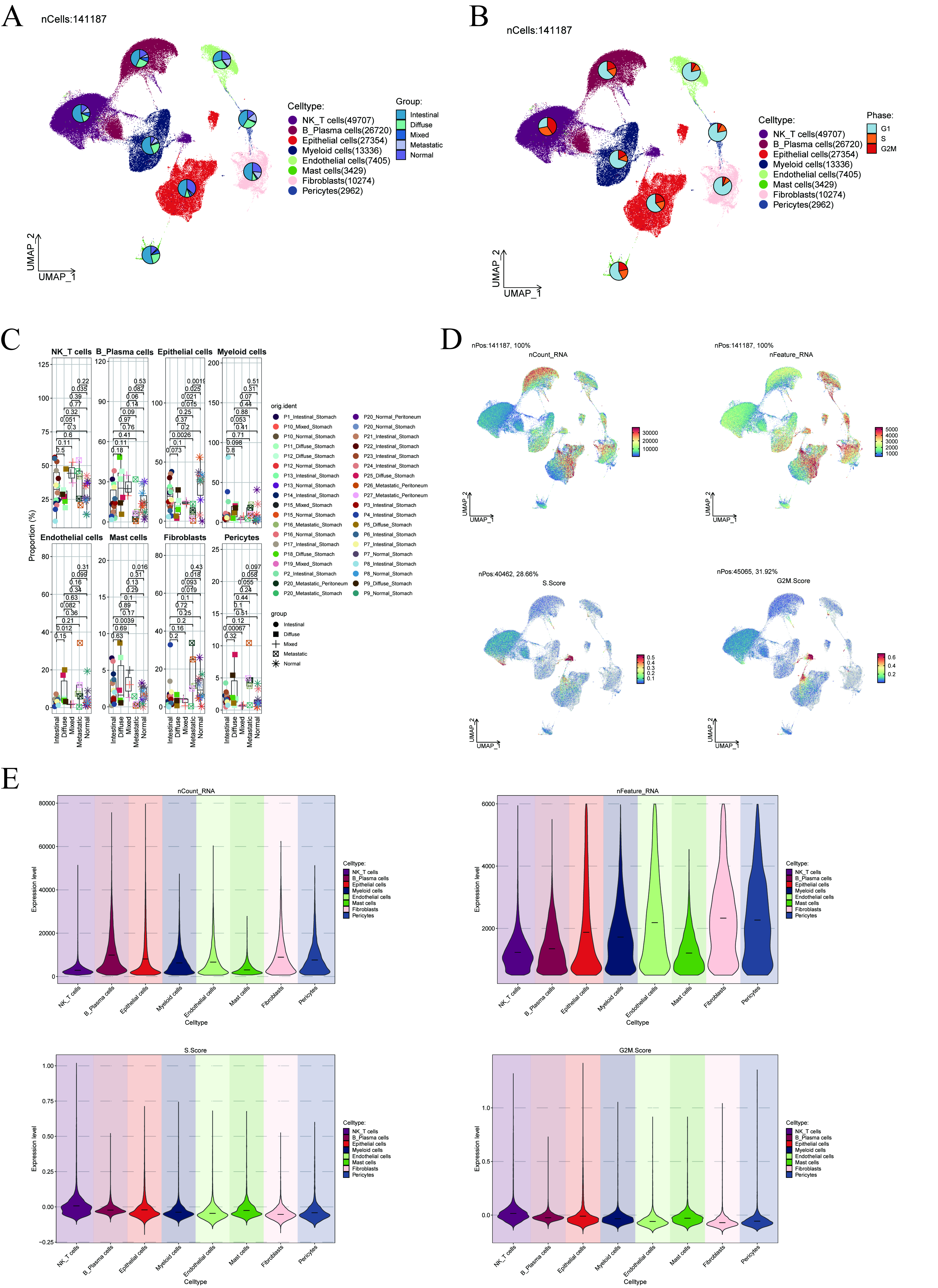

Supplement: Supplementary file 1 — Figure S1 [file JCMM-28-e18373-s006.tif]

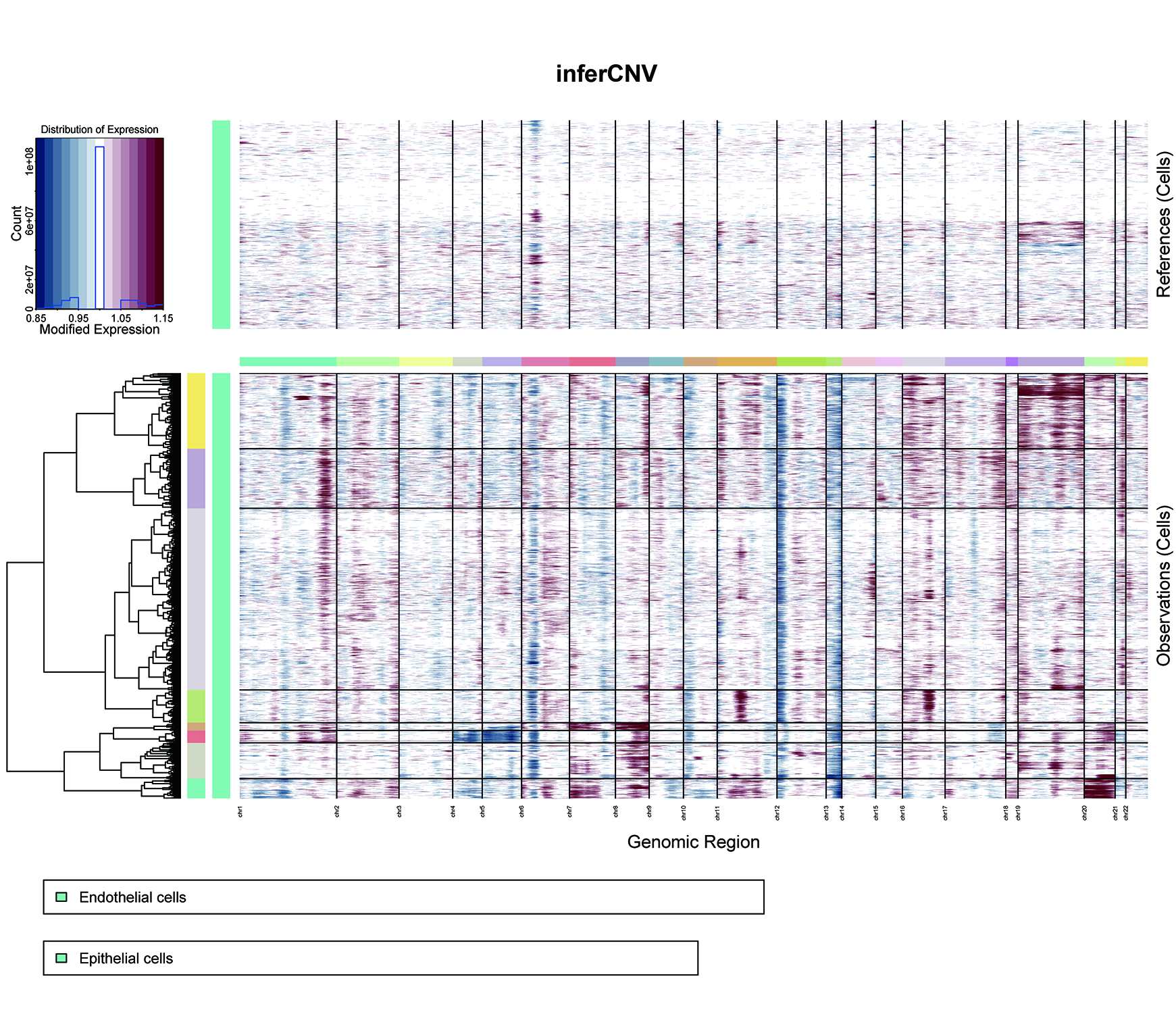

Supplement: Supplementary file 2 — Figure S2 [file JCMM-28-e18373-s004.tif]

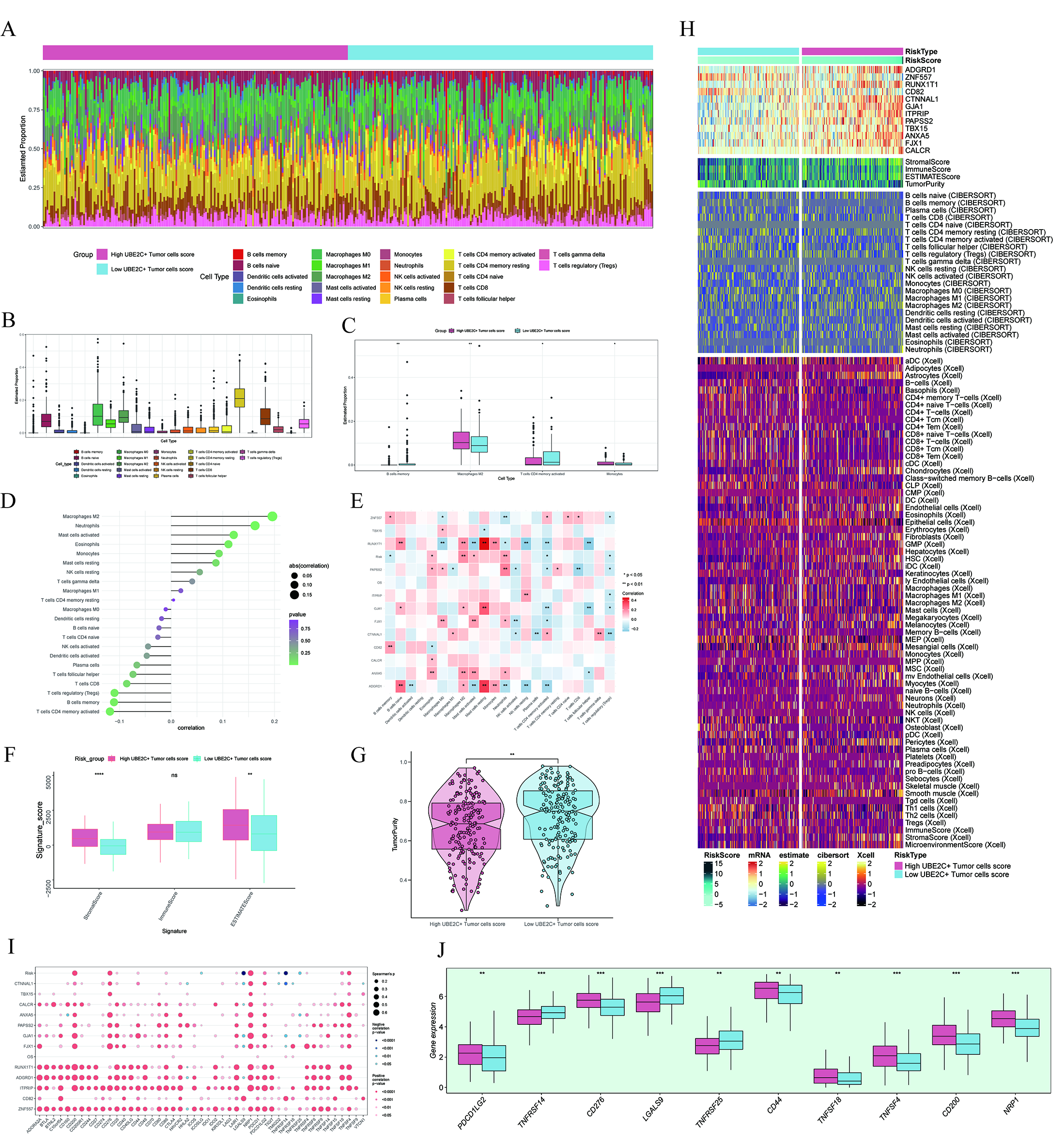

Supplement: Supplementary file 3 — Figure S3 [file JCMM-28-e18373-s001.tif]

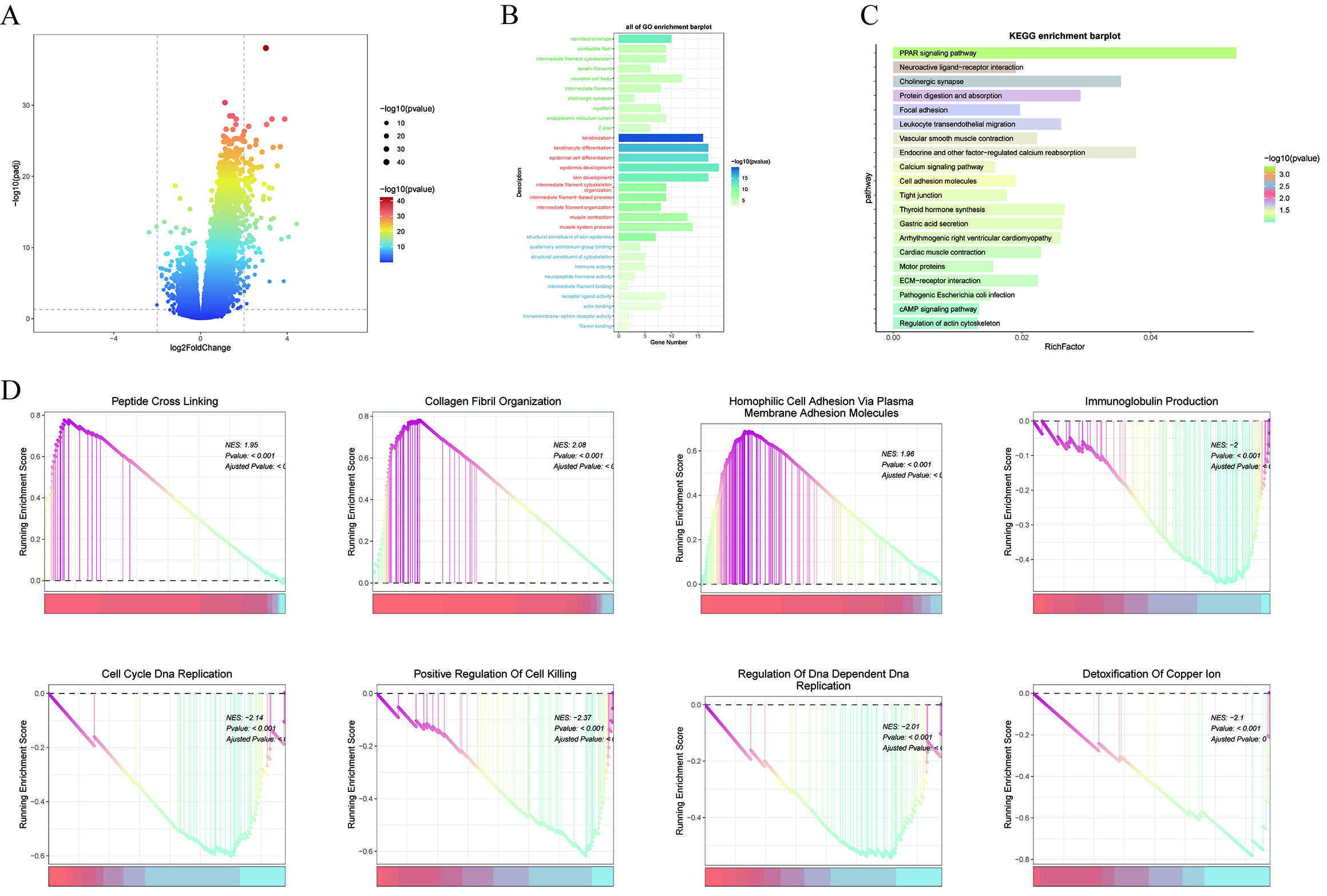

Supplement: Supplementary file 4 — Figure S4 [file JCMM-28-e18373-s003.tif]

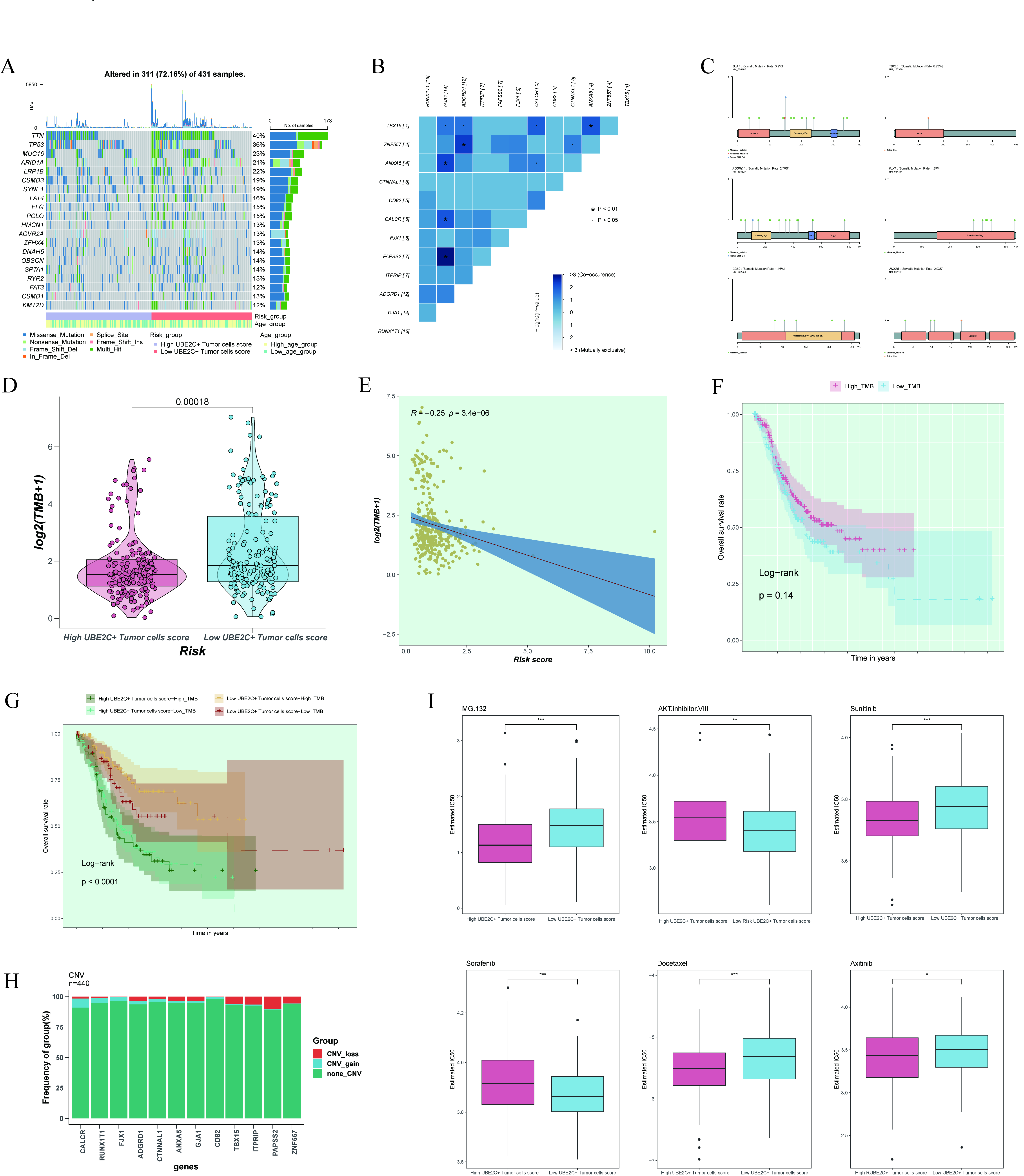

Supplement: Supplementary file 5 — Figure S5 [file JCMM-28-e18373-s008.tif]

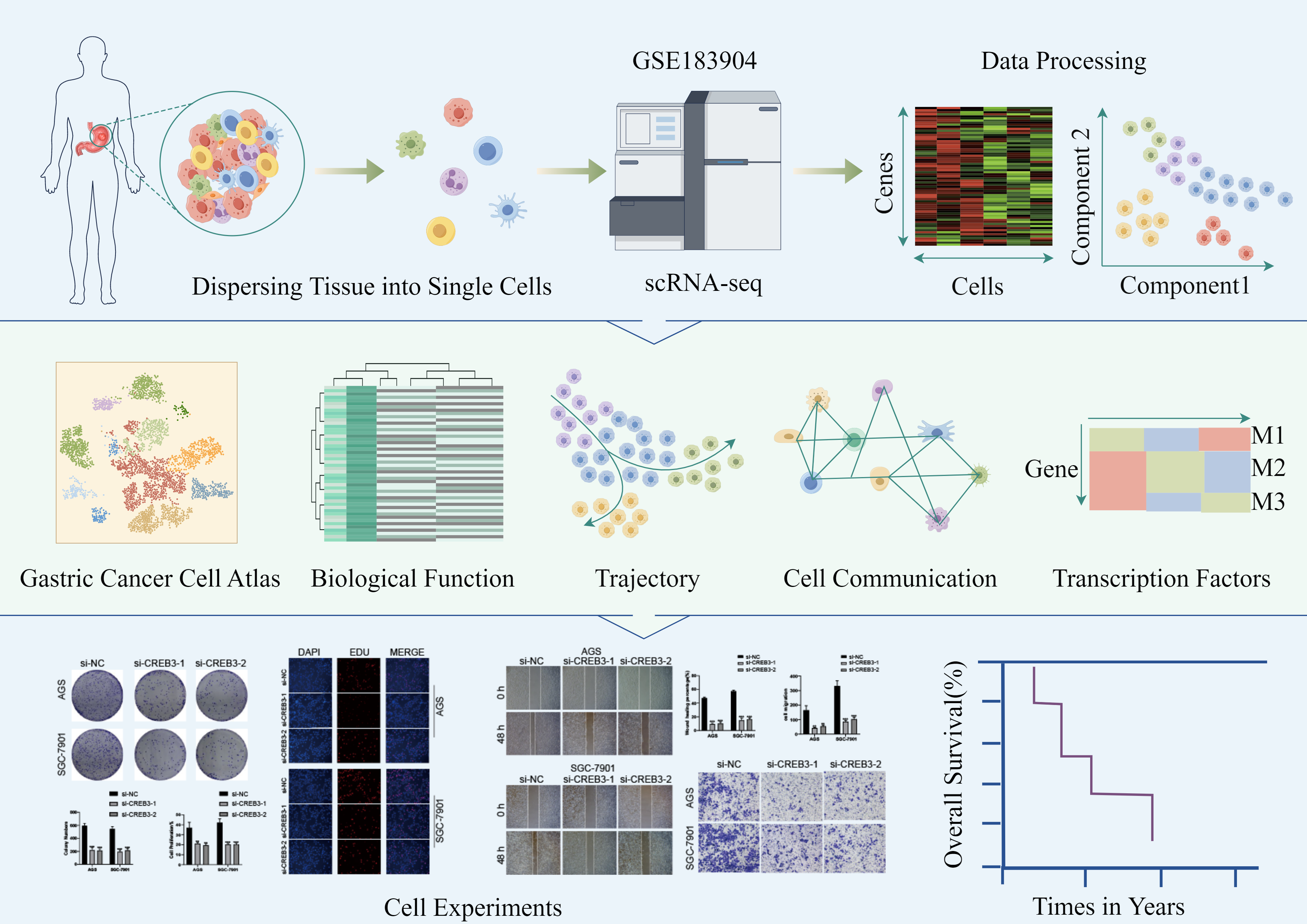

Supplement: Supplementary file 6 — Figure S6 [file JCMM-28-e18373-s005.tif]
