# Supplementary material for: Single‐cell analysis uncovers high‐proliferative tumour cell subtypes and their interactions in the microenvironment of gastric cancer
Source: J Cell Mol Med. 2024 Jun 19;28(12):e18373. doi: 10.1111/jcmm.18373 (PMC11187953; doi:10.1111/jcmm.18373)
Supplement: Supplementary file 7 — Table S1 [file JCMM-28-e18373-s002.docx]

| **Oligonucleotides** | **Nucleotide sequence (5'-3')** |
| --- | --- |
| **siRNA** |  |
| si-CREB3-1 | CUCUCACUAAGACAGAGGA |
| si-CREB3-2 | CUAGAGGAAAGUGGAGAUU |
| si-NC | UUCUCCGAACGUGUCACGU |

**Table S1. Oligonucleotides used in research**
